# c‑ALD-Grown Metal Oxide Shell Enables Distance-Independent Triplet Energy Transfer from Quantum Dots to Molecular Dyes

**DOI:** 10.1021/jacs.5c11645

**Published:** 2025-08-15

**Authors:** Marco Fabbiano, Ona Segura Lecina, Huygen J. Jöbsis, Tejas Deshpande, Skylar J. Sherman, Gordana Dukovic, Sascha Feldmann, Raffaella Buonsanti

**Affiliations:** † Laboratory of Nanochemistry for Energy, École Polytechnique Fédérale de Lausanne (EPFL), Rue de l’Industrie 17, 1951 Sion, Switzerland; ‡ Laboratory for Energy Materials, École Polytechnique Fédérale de Lausanne (EPFL), Rue de l’Industrie 17, 1951 Sion, Switzerland; § Department of Chemistry, 1877 University of Colorado Boulder, Boulder, Colorado 80309, United States; ∥ Department of Chemistry and Renewable and Sustainable Energy Institute (RASEI), University of Colorado Boulder, Boulder, Colorado 80309, United States; ⊥ Materials Science and Engineering, University of Colorado Boulder, Boulder, Colorado 80303, United States

## Abstract

Hybrid nanocomposites including quantum dots (QDs) and molecular dyes offer tunability of charge and energy transfer processes, which are attractive for applications spanning from light-emitting devices to photocatalysis and photon upconversion. Core@shell QDs have garnered attention for their ability to modulate charge and energy transfer from the core to the dye via the shell. Recent studies suggested that the core@shell QDs are less demanding in terms of the QD–dye electronic coupling requirements and that the distance dependence of transfer efficiencies may vary from what is traditionally observed in molecular systems. However, these studies remain only a few, and the mechanistic role of the shell is often unclear. Here, we synthesize QD–dye nanocomposites including CdSe@AlOx core@shell QDs, and we report the observation of a nearly distance-independent triplet energy transfer (TEnT) to polyaromatic hydrocarbon (PAH) dyes from the CdSe core of up to almost 2 nm. The use of colloidal atomic layer deposition (c-ALD) for the growth of the metal oxide shell enables tunability of the system, which is crucial to elucidate the role of the shell and the thickness dependence of the TEnT. We propose a plausible mechanism where a hole-transfer-mediated TEnT takes place and defects in the metal oxide act as intermediate states, which enable a long-range, distance-independent TEnT. The versatility of the c-ALD methodology, along with the ability of oxides to preserve the optoelectronic properties of QDs, showcases the potential of oxide shells to optimize the QD–dye interaction to achieve long-range TEnT to molecular dyes, opening new avenues for applying QDs as sensitizers in light-harvesting, emission, and conversion applications.

## Graphical abstract

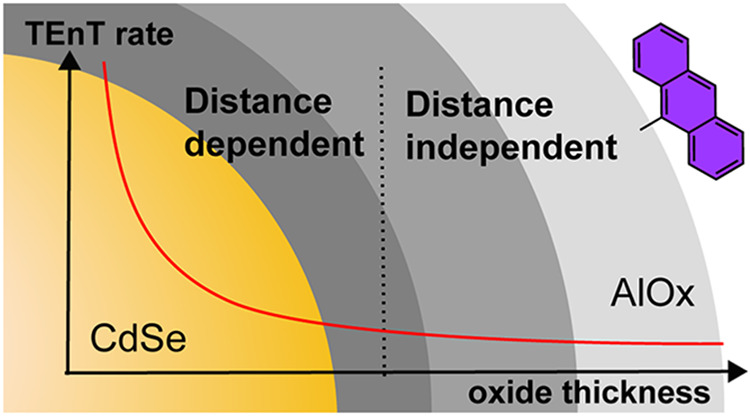

## Introduction

Colloidal quantum dots (QDs) are three-dimensionally confined semiconductor nanocrystals that are employed in a wide range of applications spanning from optoelectronics, photocatalysis, quantum information, to energy harvesting and bioimaging.^1–5^ QDs are easily processable as inks and possess advantageous features, such as large molar extinction coefficients, size-dependent electronic and optical properties, tunable surface chemistry, and properties that are complementary to those of molecular organic dyes.^6–14^ Recent years have seen increasing efforts in the combination of colloidal QDs and molecular organic dyes in nanocomposites to exploit efficient interactions between their respective excited states, enabling sensitization or harvesting of the QD excitons.^15–19^ Specifically, QD–dye nanocomposites offer the possibility of tuning charge and energy transfer processes, which is of particular interest for applications spanning from light-emitting devices to photocatalysis and photon upconversion.^20–23^ In one of the inspiring examples across the literature, nanocomposites consisting of colloidal QDs coupled with polyaromatic hydrocarbons (PAH) bound to their surface have emerged as a strategic alternative to traditional triplet sensitizers, which are based on organic dyes only.^17,24^ Contrary to molecules, QDs possess ill-defined spin quantum numbers, exhibiting closely spaced excited states and bypassing the typically larger S_1_–T_1_ energy gap of molecular dyes.^10,17,25–27^ The most common strategy to couple QDs and PAH, as well as other molecular dyes, consists of exchanging the native ligands at the QD surface for molecular dyes that have been specifically engineered with functional groups interacting with the QD surface.^24,28–31^ Then, molecular bridges between the QD core and the dye acceptors are employed to investigate the distance dependence of exciton transfer.^20,29,32^ Although this methodology has provided valuable insights, the material tunability is limited (e.g., difficulty in the synthesis of the dye with the desired functional group, challenges in tuning the number of bound dyes, and creation of trap states during ligand exchange). One alternative methodology to molecular bridges is the use of core@shell heterostructures wherein a secondary semiconductor shell acts as a spacer between the QD and the molecular dye while passivating the surface of the QD core, so that trap states are not created during ligand exchange.^33–37^ The impact of semiconductor shells on the optoelectronic properties of the core in core@shell QDs has been the subject of extensive research.^38–41^ However, the role of semiconductor shells in mediating the charge or energy transfer from the core to molecular dyes remains often elusive.^33–37,42^ The problem in data interpretation stems from the increased complexity of the exciton dynamics when the second semiconductor shell is involved in the interactions with the molecular dye along with the core.^41,43–45^ Recently, colloidal atomic layer deposition (c-ALD) has enabled the synthesis of colloidally stable core@shell comprising QDs shelled with a metal oxide.^46,47^ This method allows for the growth of a variety of metal oxides on the surface of QDs with different compositions and tunable thicknesses.^46,47^ The c-ALD-grown metal oxide shells were shown to hinder the surface ligand dynamicity and to confer increased stability to the QD core.^46,47^ In a first proof-of-concept study, PAH molecules were embedded into a hybrid organic/inorganic shell around perovskite QDs with a tunable loading; energy funneling from the core to the PAH molecules was demonstrated in this system.^48^ However, the use of these organic/inorganic shells limits the nanometer scale tunability of the distance between the nanocrystal core and the PAH molecules, which is needed to investigate distance-dependent optoelectronic phenomena.^47^

In this study, we synthesize QD@MOx/PAH nanocomposites wherein a fully inorganic oxide shell functions as a tunable spacer between the QD core and PAH acceptors to study the distance dependence of triplet energy transfer (TEnT) between QDs and PAH molecules with no spectral overlap. Taking aluminum oxide (AlOx) and 9-anthracene carboxylic acid (9ACA) as representative examples, we synthesize CdSe@AlOx/9ACA nanocomposites and demonstrate that the TEnT rates and efficiencies can be tuned by controlling the oxide thickness. Interestingly, we discover that a distance-independent process persists when thicker oxide shells are used, which we explain with the involvement of intrinsic defects within the oxide layer, enabling a long-range, distance-independent TEnT. These results provide the first evidence for a distance-independent TEnT from metal oxide-coated QDs, overcoming the short-distance limitations of the classic TEnT mechanism.

## Results and Discussion

CdSe@AlOx/9ACA nanocomposites were synthesized via c-ALD, and the tunability of this new platform was characterized via different techniques (Figures 1 and S1–S5). The shell growth is based on the c-ALD via ligand-modified metal-amide precursors (see the Methods section in the Supporting Information).^49^ The synthesis consists of two steps (Step I and Step II; Figure 1A). Step I initiates the shell growth. In the first half-cycle, a modified metal-amide precursor reacts with the native oleate ligands, resulting in a metal-amide-passivated surface and the release of oleamides via amidation. In the second half-cycle, oleic acid (OLAC) reacts with the metal-amides via proton exchange, forming a metal-oleate surface and completing one cycle. When OLAC is added, CdSe@AlOx/OLAC structures form (Step I), which will be referenced to as CdSe@AlOx for simplicity. Repeating Step I for *n* cycles enables the growth of thicker shells. Step II follows Step I to obtain the CdSe@AlOx/9ACA nanocomposites. In Step II, a mixture of OLAC and 9ACA is added to yield 9ACA molecules bound to the oxide shell with the desired loading, which is tuned by varying the OLAC:9ACA ratio. High-angle annular dark field scanning transmission electron microscopy (HAADF-STEM) and energy-dispersive X-ray spectroscopy (EDXS) maps confirm the presence of the AlOx shell (Figure S1 ). Fourier-transform infrared (FT-IR) spectroscopy and X-ray photoelectron spectroscopy (XPS) corroborate the presence of aluminum oxide, along with providing insight into the surface chemistry and the binding of OLAC and 9ACA on the c-ALD-grown AlOx (Figures S2 and S3). Diffusion-ordered spectroscopy (DOSY) provides the average increase in thickness with the number of cycles *n* (Figures 1B, S4, and S5). We used the alkene resonance of bound oleates in CdSe and CdSe@AlOx to track the evolution of the diffusion coefficients.^50–52^ The diffusion coefficient (Figure 1B, top) decreases with *n*, in line with the less steep traces of the DOSY attenuation curves (Figure S4). Consistently, the extracted hydrodynamic radius increases with *n* (Figure 1B, bottom) and can be fitted with a slope of 0.14 ± 0.02 nm/cycle, which corresponds to the average increase in shell thickness per c-ALD cycle. Based on these data, the shell thickness varies from 0.14 nm (*n* = 1) to 1.68 nm (*n* = 12) (Figure 1C). We estimated the average number of 9ACA ligands from ultraviolet–visible (UV–vis) spectroscopy following careful solvent washing (Figures 1D,E and S6). For the same number of cycles, the number of bound 9ACA per QD increases from around 6 to 38 when increasing the 9ACA:OLAC molar ratio from 0.25:1 to 4:1 in Step II (Figure 1D). The optical absorbance of 9ACA in CdSe@AlOx/9ACA, referenced to the corresponding CdSe@AlOx, increases consistently with increasing the 9ACA loading (Figure 1E). Comparison with ligand-exchanged CdSe/9ACA as a control sample evidenced the advantage provided by the c-ALD approach in achieving superior control on loading tunability (Figure 1D) as well as increased dye loading when the same 9ACA amount is added in solution (Figure S6).

**Figure 1:**
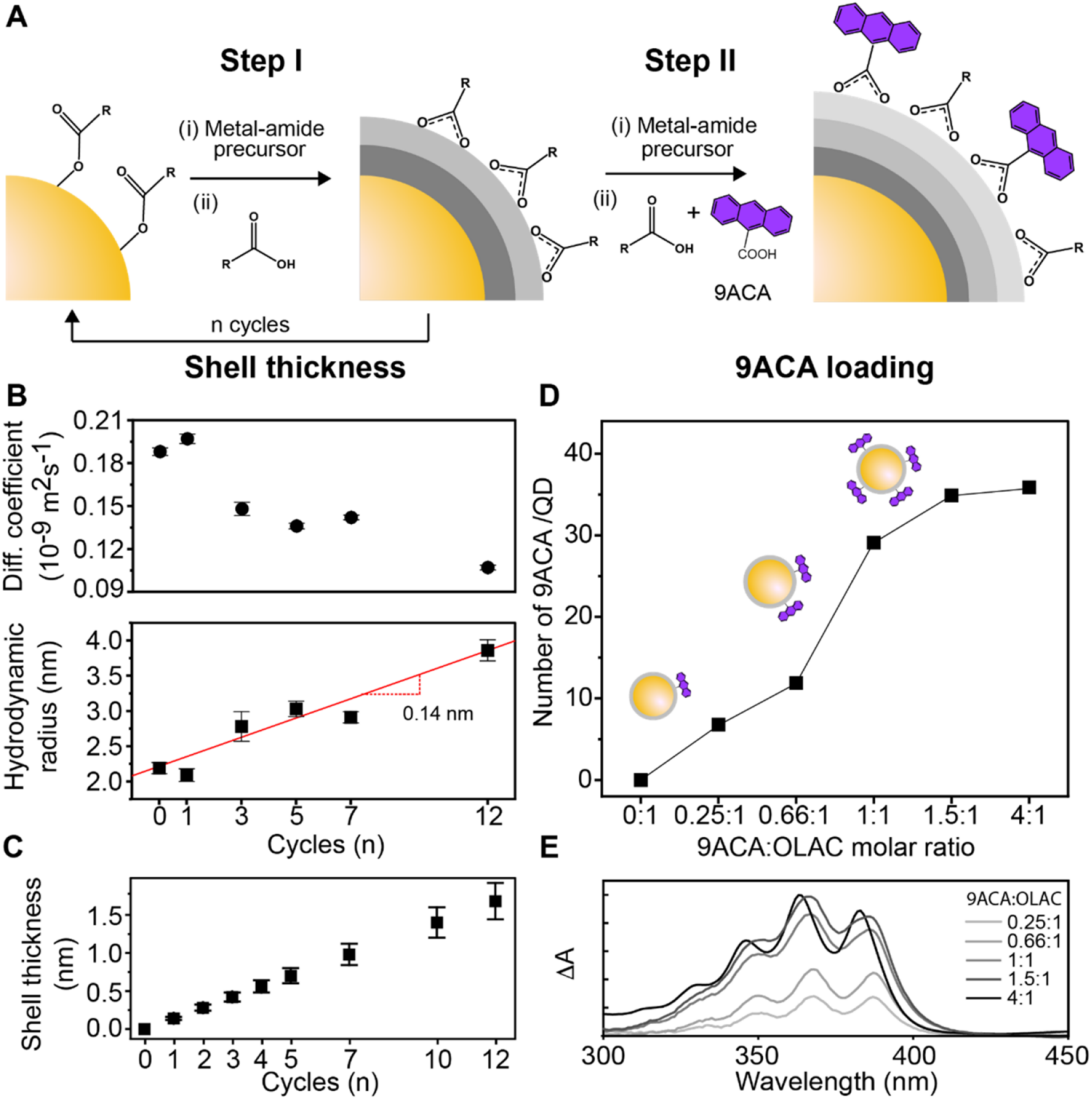
Synthesis and tuning of shell thickness and ligand loading in CdSe@AlOx/9ACA nanocomposites. (A) Schematic representation of the c-ALD process to synthesize CdSe@AlOx (Step I) and CdSe@AlOx/9ACA (Step II). (B) Diffusion coefficients (top) and hydrodynamic radii (bottom) from DOSY estimated from the alkene resonance of bound oleates. (C) Average increase in shell thickness with the number of cycles estimated based on the data in panel (B). (D) Average number of 9ACA per QD at different 9ACA:OLAC molar ratios for the same shell thickness (*n* = 1). The solid line connecting the data points helps the viewer to follow the trend. (E) Absorption spectra of CdSe@AlOx/9ACA for *n* = 1 at different 9ACA:OLAC molar ratios which were referenced to the absorption spectrum of CdSe@AlOx for the same number of cycles. The intensity of the 9ACA optical features increases along with the increased 9ACA:OLAC ratio utilized in Step II.

Having successfully developed tunable CdSe@AlOx/9ACA nanocomposites, we investigated the optical properties of this new platform. First, we measured UV–vis absorption and PL emission for varying *n* (i.e., shell thickness) with the same 9ACA loading for all samples (Figures 2 and S7). The *E*_1S‑1S_ absorption peak and the band edge emission of CdSe@AlOx are centered at 525 and 540 nm, respectively, and show no significant shift or broadening with the number of cycles (Figure 2A, dashed lines), indicating that the average core size is retained and the exciton wave function is confined within the core. This result is consistent with the growth of a shell with a wider band gap.^39,53,54^ Furthermore, the CdSe@AlOx/9ACA absorption profiles display the characteristic absorption of 9ACA in octane ranging from 400 to 320 nm (Figure 2A, solid lines), confirming the presence of the latter in all of the samples. The CdSe@AlOx/9ACA emission profiles (Figure 2B, solid lines) evidence quenching of the band edge emission compared to CdSe@AlOx (Figure 2B, dashed lines). The quenching is more pronounced for thinner shells. The CdSe emission gradually recovers as the shell thickness increases; yet, CdSe@AlOx/9ACA does not fully reach the emission of the bare CdSe@AlOx even for the thicker shells, suggesting that 9ACA sensitization still occurs. Both the energetic band diagram and the literature on CdSe/9ACA support the occurrence of TEnT via a Dexter-like mechanism from CdSe to the 9ACA (Figure S8).^17,24,29,36^ Thus, we assume this process to be the dominant quenching mechanism in the CdSe@AlOx/9ACA nanocomposites.

**Figure 2:**
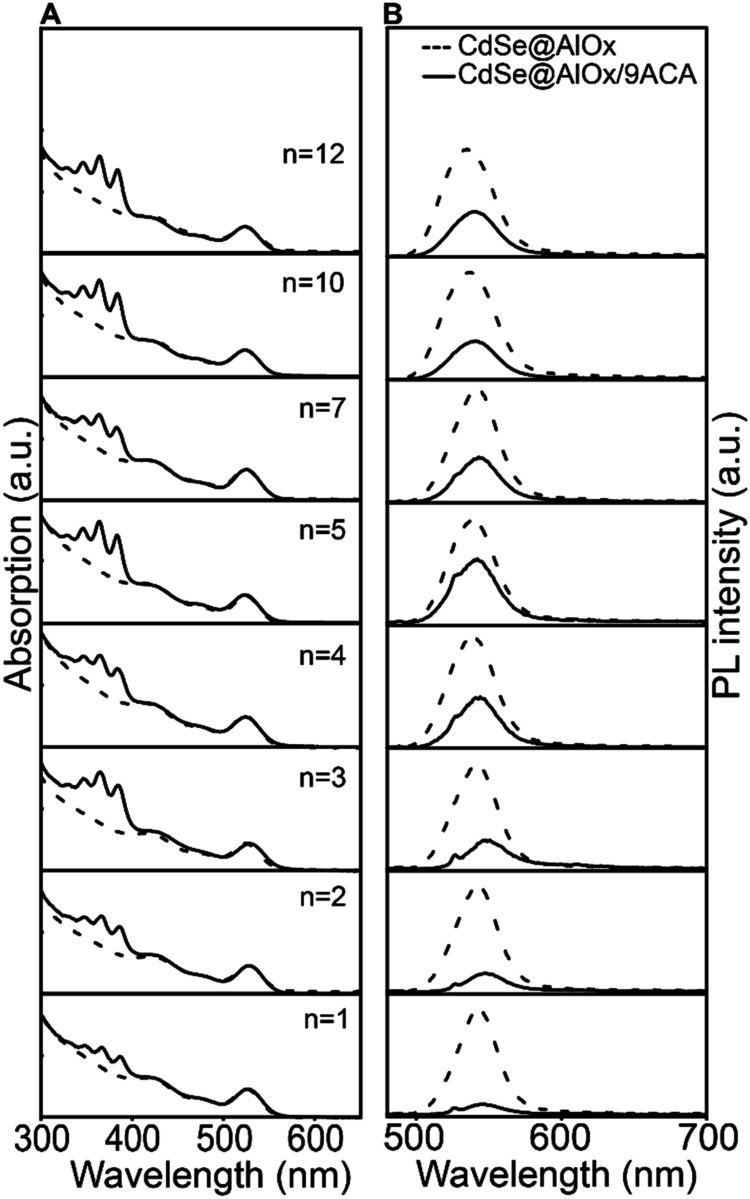
Optical properties of the CdSe@AlOx/9ACA with variable AlOx shell thickness. (A) UV–vis absorption spectra and (B) PL emission spectra of CdSe@AlOx (dashed lines) and CdSe@AlOx/9ACA (solid lines). All emission spectra are acquired with excitation at 455 nm to excite CdSe while avoiding direct excitation of 9ACA. These measurements were performed on 2.7 nm of CdSe QDs with 9ACA loadings in a range of 20–25 molecules per QD. This loading was chosen for the optimal colloidal stability of all of the samples.

To probe the kinetics of PL quenching as a function of the donor–acceptor distance (i.e., shell thickness) in CdSe@AlOx/9ACA, we conducted time-resolved PL (TRPL) decay measurements by exciting at 455 nm to populate the excited states of CdSe (Figures 3 and S9–S11). In CdSe@AlOx, slower PL decays are observed as the number of cycles increases (Figure 3A) and, concomitantly, the average lifetime increases from around 60 to more than 80 ns as the AlOx grows thicker (Figure 3B, empty squares), suggesting a longer-lived excited state of the CdSe core as the surface defect states are passivated by the metal oxide. The average lifetimes are consistently shortened in the presence of the 9ACA (Figure 3B, filled squares). The most pronounced quenching corresponds to the CdSe QDs functionalized with 9ACA through ligand exchange (*n =* 0). Then, the average lifetime almost linearly approaches that of CdSe@AlOx up to a thickness of 0.7 nm (*n* ≤ 5). These results further support the hypothesis that the sensitization of 9ACA is less efficient as the donor–acceptor electronic coupling weakens, as expected from a Dexter-like mechanism.^55,56^ Yet, the average lifetime remains almost constant beyond 0.7 nm (*n* > 5), indicating that sensitization of 9ACA still takes place with the relatively thick-shelled.

**Figure 3:**
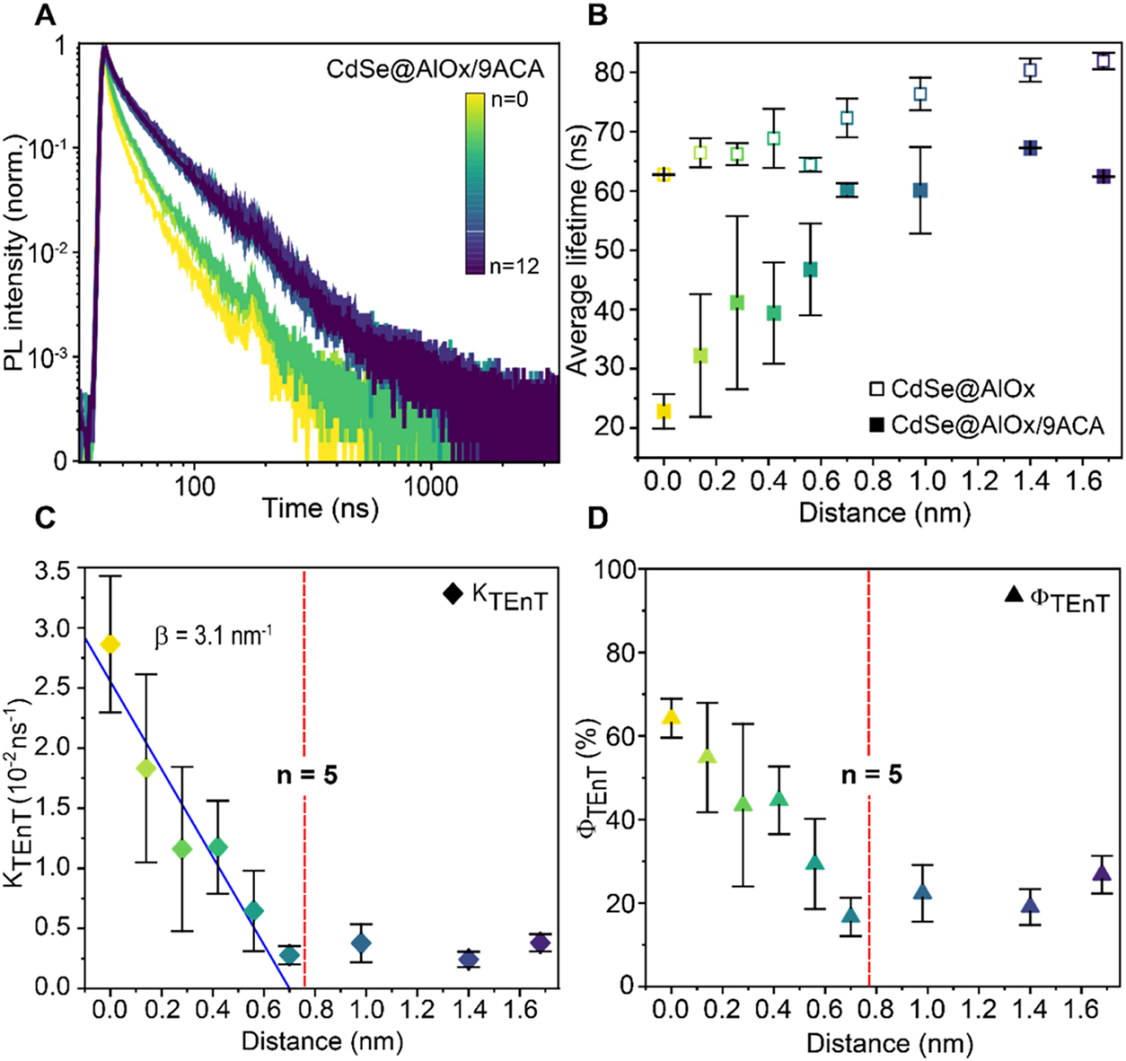
Time-resolved PL (TRPL) decays and modeling of decay rate as TEnT. (A) TRPL of CdSe@AlOx with different shell thicknesses at a 455 nm excitation wavelength. A graded-color legend is used to indicate the number of cycles. (B) Average lifetimes as a function of the distance for CdSe@AlOx (empty squares) and CdSe@AlOx/9ACA (filled squares). (C) Calculated rates of TEnT as a function of the shell thickness. The red dashed line separates the distance-dependent and distance-independent regimes, corresponding to *n* = 5 and a shell thickness of around 0.7 nm. The blue line represents the linear regression, and the fitted damping coefficient β is shown. (D) Calculated TEnT efficiencies as a function of the shell thickness. The same red dashed line is used to separate the two regimes at *n* = 5.

To gain further insight, we modeled the PL decay traces to extract the rates and efficiencies of TEnT as a function of shell thickness (Figures 3C,D and S9). We used eq 1 to determine the rates of TEnT, *k*_TEnT_ ^57^

1 $$k_{\mathrm{TEnT}}=\frac{1}{\left\langle \tau_{\mathrm{CdS}e_{n}+9\mathrm{ACA}}\right\rangle }-\frac{1}{\left\langle \tau_{\mathrm{CdS}e_{n}}\right\rangle }$$

where ⟨τ_CdSe*_n_* +9ACA_⟩ is the average lifetime of samples with bound 9ACA ligands and ⟨τ_CdSe*_n_*_⟩ is the average lifetime of samples without acceptors. In the absence of other decay pathways, the difference can be interpreted as the rate of TEnT. We can directly compare the rates between the samples with different shell thicknesses because the 9ACA loading is relatively constant at 20–25 molecules per QD in all of them. The rate *k*_TEnT_ decreases with a shell thickness of up to 0.7 nm (*n* = 5) and remains constant for thicker shells (Figure 3C). The efficiency follows the same trend and reduces from ∼65 to ∼20% up to 0.7 nm (*n* = 5) and remains constant for thicker shells (Figure 3D). The decay behavior up to *n* = 5 is consistent with a Dexter-like TEnT mechanism. Here, we extracted the damping coefficient β, a measure of the donor–acceptor electronic coupling as a function of distance, which provides further insight into the TEnT mechanism.^58–60^ The linear fitting for *n* ≤ 5 yields an experimental β of 3.1 ± 0.3 nm^–1^ (Figures 3C and S10). We further model our PL decay data using a Poisson-distributed population model to estimate the single-acceptor rate constants of TEnT, for which we find a very similar trend and an experimental β of 4.9 ± 0.6 nm^–1^ (Figures S11 and S12). The experimentally determined β agrees with a sequential hole-transfer-mediated mechanism rather than a concerted tunneling mechanism, for which higher β values are expected.^20,34,36,42,56^ A sequential hole-transfer-mediated mechanism has been recently observed for other core@shell structures, specifically CdSe@ZnS functionalized with 9ACA molecules and when molecular conjugated bridges are used to separate the CdSe core from the acceptor ligand.^20,29,34,36^ This literature provides strong support for our hypothesis. However, the finding that a distance-independent phenomenon takes place beyond a 1 nm separation in CdSe@AlOx/9ACA is unexpected.

To confirm that a net TEnT occurs across the oxide shell, we performed ultrafast transient absorption (TA) spectroscopy experiments (Figures 4 and S13). Similarly to the TRPL experiment, we populated the excited state of the QD core by exciting it resonantly with the band-gap energy (i.e., 525 nm). The recovery of the ground-state bleach (GSB, centered at 515 nm; green-shaded area in Figure 4A) and photoinduced absorption (PIA) associated with the QD core is accompanied by a gradual increase of a PIA feature characteristic for the 9ACA triplets (at 430 nm; blue-shaded region in Figure 4A) The ingrowth of the triplet PIA feature for *n* = 0 (Figure 4B) follows similar kinetics as previously observed for TEnT in similar systems, with a time constant of 8.5 ns (which corresponds to a rate of around 0.11 ns^–1^), which confirms triplet sensitization (Figure S13).^20,36,42^ Passivation of the QDs upon growing the oxide shell (*n* = 4) prolongs the lifetime of the QD excited state (Figure 4C,D). However, the triplet feature is still distinguishable from the QD features, confirming TEnT through the shell. Upon further increasing the shell thickness (*n* = 7), the triplet feature cannot be separated from the long-lived QD PIA feature around 430 nm (Figure 4E,F) within the time resolution of the ultrafast TA experiments.

**Figure 4:**
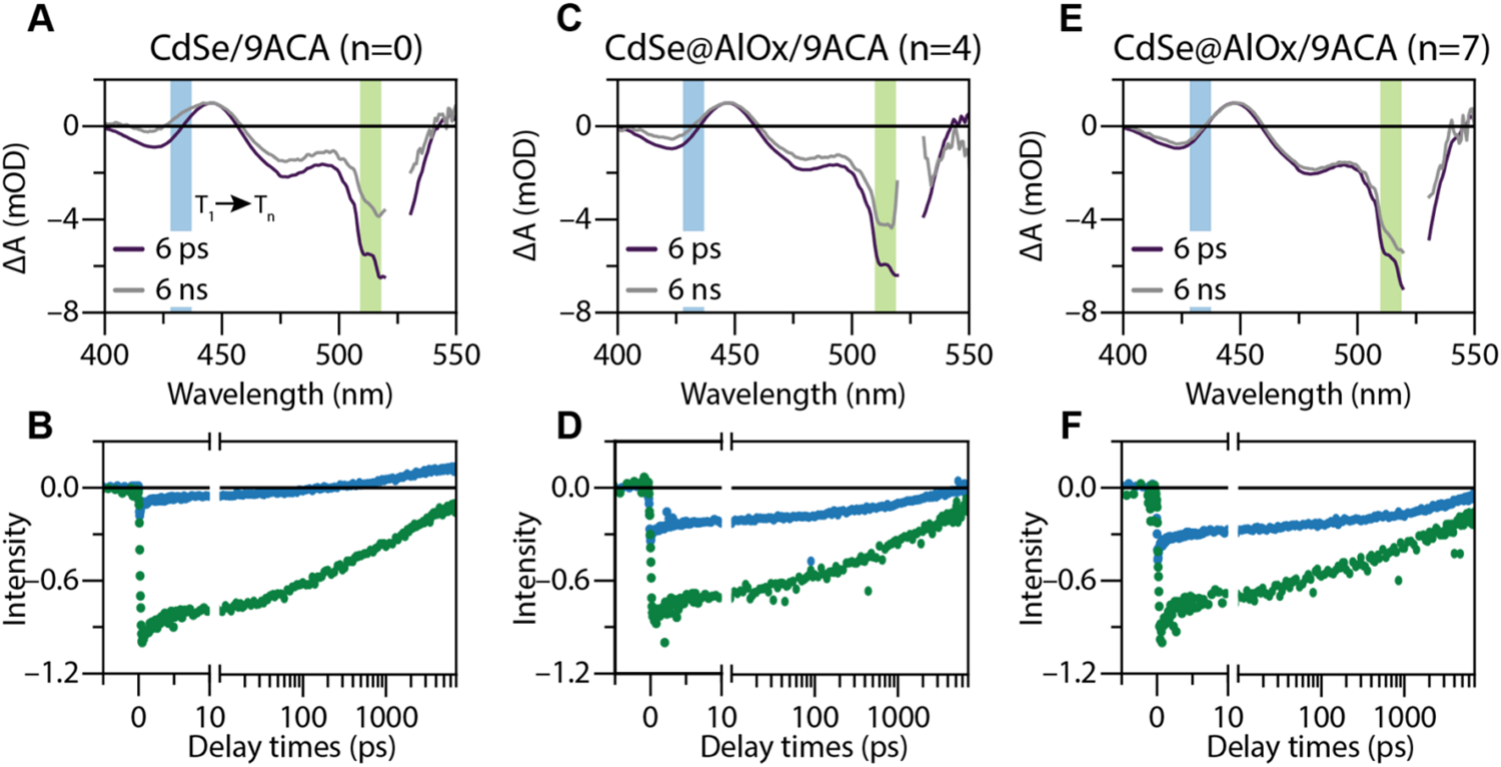
Ultrafast transient absorption spectroscopy confirming the triplet sensitization in the distance-dependent regime. (A, C, E) Differential absorption spectra of 6 ps and 6 ns after exciting the QD core using a 525 nm pump pulse (ca. 100 fs long) and (B, D, F) corresponding ingrowth kinetics of the PIA, integrated at 430 nm for (A, B) bare CdSe/9ACA QDs (*n* = 0), (C, D) CdSe@AlOx/9ACA for *n* = 4, and (E, F) CdSe@AlOx/9ACA for *n* = 7. The broadening of the PIA signal at 6 ns upon increasing the AlOx thickness (*n* = 4) and the ingrowth of the triplet feature suggest that TEnT occurs across the shell. No triplet feature can be distinguished for *n* = 7 in the timescale of these measurements due to the increased charge carrier lifetime.

Therefore, we performed nanosecond TA spectroscopy experiments to evaluate the TEnT for *n* = 7 at longer time scales (Figure 5). The comparison of the differential absorption spectra of the sample without and with 9ACA (Figure 5A,B, respectively) evidences a broadening of the PIA feature around 430 nm. This broadening, along with an ingrowth of the triplet PIA feature in the decay dynamics (Figure 5C), confirms the TEnT in CdSe@AlOx for *n* = 7 occurring at longer time scales. We expect samples with *n* = 10 and *n* = 12 to exhibit even longer charge carrier lifetimes and, thus, to require TA measurements in the millisecond regime.

**Figure 5:**
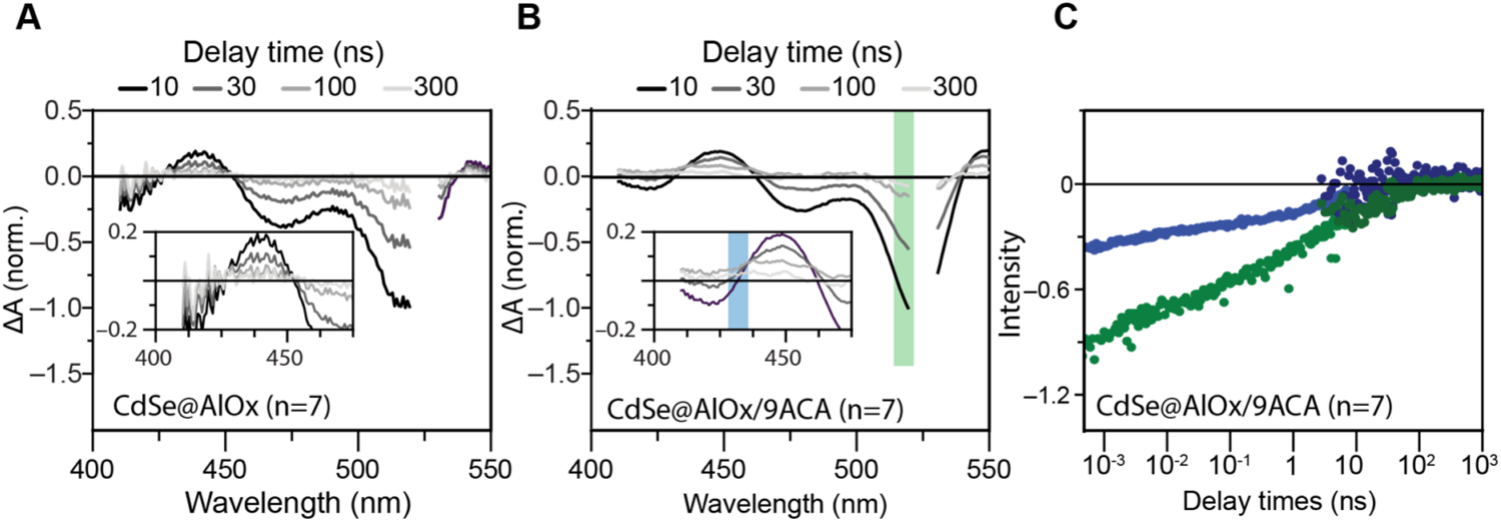
Nanosecond transient absorption spectroscopy confirming the triplet sensitization in the distance-independent regime. (A, B) Spectral slices of the differential absorption spectra at 10, 30, 100, and 300 ns after exciting the QD core using a 525 nm pump, with no 9ACA ligands (A) and with **9**ACA ligands (B). (C) Decay dynamics of the ground-state bleach of the QD core at 515 nm (green trace) and the PIA feature for the QD core with 9ACA ligands at 430 nm (blue trace) are compared. These data confirm a triplet feature for *n* = 7 detectable in the time scale of hundreds of nanoseconds.

Having provided evidence of a net TEnT across the oxide shell, an overall picture of the energy transfer mechanism in the CdSe@AlOx/9ACA composites at different AlOx shell thicknesses emerges (Figure 6). Upon light absorption (455 nm), CdSe quickly populates its triplet-like state thanks to the ill-defined spin quantum numbers and small S_1_–T_1_ splitting, enabling the triplet-like exciton to transfer to the T_1_ state of 9ACA. The β value indicates a hole-transfer-mediated mechanism rather than a concerted Dexter mechanism where the unsplitted exciton transfers. In the presence of a relatively strong CdSe-9ACA electronic coupling (shell thickness ≤ 0.7 nm), the electrons can directly sensitize the T_1_ state of 9ACA (solid red arrow). For thicknesses greater than 0.7 nm (*n* > 5), the weak electronic coupling would not allow for the direct sensitization of 9ACA. However, a distance-independent TEnT is observed up to almost 2 nm. Based on our findings, we propose that defects of the AlOx shell act as intermediate states. Being the c-ALD a low-temperature process, the growth of amorphous AlOx via this method most likely yields the unintentional formation of defects such as oxygen vacancies or residual carbon, which will fall within the band gap of the oxide.^49,61–66^ These intermediate states extend the charge carrier lifetime by allowing the electrons to move across the oxide over longer distances in a multistep fashion (dashed red arrows). Eventually, a distance-independent TEnT to 9ACA is established.

**Figure 6:**
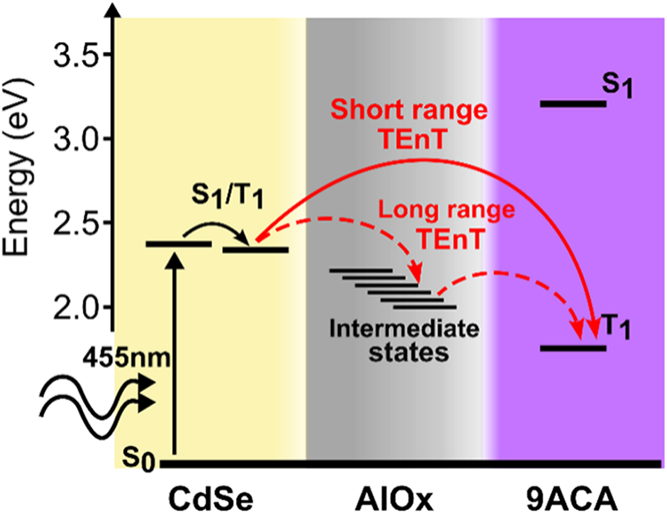
Proposed distance-dependent mechanisms of TEnT across the oxide shell in CdSe@AlOx/9ACA nanocomposites. TEnT occurs through direct sensitization of the 9ACA T_1_ state (solid red arrow) for thinner oxide shells (i.e. up to 0.7nm). Defect states act as intermediate states for the sensitization of 9ACA (dashed red arrows) for thicker oxide shells to generate a long range distance-independent TEnT.

## Conclusions

In conclusion, we synthesized c-ALD-grown QD@MOx/PAH nanocomposites and proposed them as a new tunable platform for the triplet energy transfer between QDs and PAH molecules without spectral overlap. Taking CdSe@AlOx/9ACA as a representative example, we demonstrated that the oxide shell thickness tunes the TEnT rates and efficiencies. We determined a damping coefficient β of 3.1 nm^–1^, which aligns well with a sequential hole-transfer-mediated mechanism. To the best of our knowledge, this study is the first report of a Dexter damping coefficient for metal oxide-coated QDs. Interestingly, we found that a nearly distance-independent process dominates for thicker oxide shells, which we attributed to a net TEnT via transient absorption measurements. Finally, we proposed a plausible mechanism where the intrinsic defect states within the metal oxide act as intermediate states, enabling electrons to move across the oxide shell, eventually yielding a long-range TEnT. The observation of a distance-independent TEnT across an inorganic oxide shell is unprecedented up to now and stimulates future studies on both fundamental physical chemistry (e.g., photon upconversion) and applications.

Overall, this work highlights the potential of metal oxide-coated QDs to enable long-range TEnT while preserving the core optoelectronic properties, further expanding the scope of QDs for light-driven applications based on long-range triplet exciton transport.

## Supplementary Material



## Data Availability

Experimental raw data are openly available in Zenodo at 10.5281/zenodo.15845734.

## Notes

Supporting methods; additional experimental considerations; additional HAADF-STEM–EDXS, XPS, FT-IR, and NMR; and additional transient absorption and spectroscopy data (PDF)
