# Supplementary material for: c‑ALD-Grown Metal Oxide Shell Enables Distance-Independent Triplet Energy Transfer from Quantum Dots to Molecular Dyes
Source: J Am Chem Soc. 2025 Aug 15;147(34):31409–16. doi: 10.1021/jacs.5c11645 (PMC12395480; doi:10.1021/jacs.5c11645)
Supplement: Supplementary file 1 [file ja5c11645_si_001.pdf]

Supplementary Information

c-ALD-grown Metal Oxide Shell Enables Distance-Independent Triplet Energy Transfer from Quantum Dots to Molecular Dyes

Marco Fabbiano[1], Ona Segura Lecina[1], Huygen J. Jöbsis[2], Tejas Deshpande[2], Skylar J. Sherman[3], Gordana Dukovic[3], Sascha Feldmann[2], Raffaella Buonsanti[1*]

1. Laboratory of Nanochemistry for Energy, École Polytechnique Fédérale de Lausanne (EPFL), Rue de l'industrie 17, 1951 Sion, Switzerland.
2. Laboratory for Energy Materials, École Polytechnique Fédérale de Lausanne (EPFL), Rue de l'industrie 17, 1951 Sion, Switzerland.
3. Department of Chemistry, University of Colorado Boulder, Boulder, Colorado 80309, United States

*Corresponding author: raffaella.buonsanti@epfl.ch

Materials and Instruments

Chemicals

9-antrachene carboxylic acid (9ACA, $C_{14}H_9COOH$, 99%) was purchased from Fluorochem. Methanol (MeOH, CH_3OH, anhydrous, 95%), was purchased from ThermoScientific. Dibromomethane (CH_2Br_2, 99%) was purchased from Acros. Cadmium Oxide (CdO, 99.99%), Didodecylamine (dda, $[CH_3(CH_2)_{11}]_2NH$, 97%), Ethanol (EtOH, CH_3CH_2OH, anhydrous, 95%), 1-Octadecene (1ODE, $CH_3(CH_2)_{15}CH=CH_2$, 90%), Octane (OT, C_8H_{18}, anhydrous, 95%), Oleic acid (OLAC, $CH_3(CH_2)_7CH=CH(CH_2)_7COOH$, 90%), Selenium (Se, 99.99%), Toluene (tol, $C_6H_5CH_3$, anhydrous, >96%), Toluene-d8 (tol-d8, $C_6D_5CD_3$, 99.5 atom%D), Tris(dimethylamino)aluminum (TDMA-Al, $Al[N(CH_3)_2)]_3$, 98%), were purchased from Sigma Aldrich.

Instruments

Optical absorption: Measured using a PerkinElmer Lambda 950 spectrophotometer, equipped with a deuterium (ultraviolet range) and tungsten lamp (visible and infrared range). A PMT and InGaAs detector was used.

Photoluminescence (PL) and Time-Resolved PL (TRPL) measurements. PL and TRPL measurements were conducted using a JY Horiba Fluorolog 3 equipped with a 450 W xenon arc lamp and a PMT detector. A Horiba Jobin Yvon Fluorolog 3 instrument was employed to acquire time-resolved PL. A Horiba nanoLED with an excitation wavelength of 455 nm was employed to excite the QDs core. The samples are prepared in airtight 1 cm cuvettes by adding a small volume of the sample to 2 mL octane after baseline correction. The added volume is such that an absorption of around 0.2 is obtained at the QDs E1S peak position.

Femtosecond transient absorption. The fs-TA experiments were performed using a setup based on modules supplied by Light Conversion, with a 1030 nm seed laser (PHAROS, Light Conversion, Yb:KGW lasing medium, 340 mW pulse energy, 50 fs duration, operated at 5 kHz repetition rate). The pump beam (525 nm, ca. 900 μm in diameter) was generated using an optical parametric amplification (OPA) unit (ORPHEUS, Light Conversion), which generated a signal (525 nm) and an idler from the fundamental 1030 nm seed (fluence 2.82×10^{13} cm^{-2}). The probe beam (ca. 200 μm in diameter) was generated from the second harmonic of the fundamental using supercontinuum generation in a sapphire crystal. The probe generated from the 515 nm second harmonic spanned from ca. 380–550 nm. The pump-probe delay was

controlled over a range of 7 ns by changing probe path length *via* a multi-pass delay stage, and the pump was passed through an optical chopper (75 Hz), where a beamsplitter/photodiode combination was used to divide and sort measurements into pumped and unpumped. The probe beam was passed into a grating spectrograph (Andor Kymera 193i) and recorded using a Si NMOS photodiode array detector (256 pixels).

Nanosecond transient absorption. The ns-TA experiments were performed using the same excitation source used for the fs-TA measurements. The fundamental laser (PHAROS, Light Conversion) was operated at 2 kHz and the 525 nm pump beam was created using the OPA (ORPHEUS, Light Conversion) (fluence 2.80×10^{13} cm^{-2}). The white light pulses were generated using a LEUKOS super continuum light source (200–2400 nm, 200 mW, < 1ns) operating at 2 kHz. The pump on/off probe spectra were also recorded using the same detector as for the fs-TA experiments mentioned above.

X-ray Photoelectron Spectroscopy (XPS). XPS measurements were recorded using a Kratos Analytical instrument, using the monochromatic Kα X-ray line of an Al anode. The pass energy was set to 20 eV with a step size of 0.1 eV. The samples were prepared by drop-casting nanocrystal films onto clean Si substrates. The samples were electrically insulated from the sample holder and charges were compensated. Curve fitting was performed using CasaXPS software. Spectra were referenced at 284.8 eV using the C–C bond of the C1s orbital.

Nuclear Magnetic Resonance (NMR) spectroscopy. Solution NMR measurements were recorded on a Bruker Avance III HD 400 MHz 9.4 T spectrometer equipped with a BBFO liquid probe. One-dimensional (1D) 1H spectra were acquired using a standard pulse sequence from the Bruker library. The quantitative 1D 1H spectra were recorded with 90 s relaxation delay (d1) to allow full relaxation of the internal standard (std) (dibromomethane, T1 ≈ 19 s). The concentration of the species of interest (*x*) was calculated using eq.1, where C corresponds to concentration, A to the integral area and N number of nuclei producing the signal. Integration values were obtained by fitting in TopSpin.

$$C_x = \frac{A_x}{A_{std}} \cdot \frac{N_{std}}{N_x} \cdot C_{std} \quad (1)$$

The density of bound ligands on the QDs surface was calculated assuming that (i) QDs possess a spherical shape corresponding to a surface area $A=4\pi r^2$ and that (ii) the concentration of QDs is calculated from UV-VIS absorption spectra. Preparing an NMR tube with a known QDs concentration, it is possible to calculate the OLAC concentration in the tube. It is therefore

trivial to extrapolate the value of OLAC/QD and OLAC/nm^2. All the samples are prepared using Toluene-d8.

Diffusion-ordered spectroscopy (2d-DOSY). 2d-DOSY was performed with a pulse field gradient spin-echo PFGSE decay (pulse sequence *ledbpgp2s* from the Bruker library). The gradient strength was varied from 5 to 90 % of the maximum strength using a smoothed squared gradient shape. The diffusion coefficient (D) is extracted from the hydrogen alkene resonance of bound OLAC ligands, enabling the calculation of the average hydrodynamic radius (r_H) employing the Stokes-Einstein equation (eq.2)

$$D = \frac{K_B T}{6\pi\eta r_H} \tag{2}$$

Where K_B is the Boltzmann constant, T the temperature expressed in Kelvin (298 K) and η the viscosity of the solvent. For Toluene-d8 $\eta = 0.56 \cdot 10^{-3} Pa \cdot s$.

High-angle annular Dark-field scanning TEM (HAADF-STEM) and Energy Dispersive X-ray (EDXS). These measurements were performed using a FEI Tecnai Osiris TEM in scanning mode at an acceleration voltage of 200 kV. This microscope is equipped with a high brightness X-FEG gun, and four 4 windowless Super-X silicon detectors for EDXS. The EDXS data was processed using the Bruker Esprit software (version 1.9).

Methods

Synthesis of CdSe QDs

CdSe QDs were prepared by a modified procedure reported by Flamee et al.[1] A 50 mL three-necked round flask is loaded with CdO (0.514 g, 4 mmoles, 1 eq), oleic acid (OLAC, 5.1 mL, 16 mmoles, 4 eq) and 1-Octadecene (ODE, 10 mL). The reaction mixture is heated to 230 °C under nitrogen flow to dissolve the red CdO in ODE, forming the Cd(oleate)$_2$ complex. Afterwards, the solution is left to cool down and it is degassed at 100 °C for 30 minutes. The heterogeneous Se-ODE 2M dispersion precursor is prepared by adding Se powder (1.58 g, 20 mmoles, 5 eq) to 10 mL ODE in a vial at room temperature and the resulting dispersion is left stirring under nitrogen flow until the hot injection is performed. No attempt to dissolve Se powder was made. To initiate the reaction, 1 mL of Se-ODE precursor is swiftly injected using a 10 mL syringe and a thick needle. The hot injection is performed at 260 °C under nitrogen flow and the black color of Se powder disappears upon injection. The growth takes place for 0.1 to 1 minute and the colorless solution turns from yellow to orange to red depending on the size of CdSe QDs. The crystal growth is quenched by the injection of 10 mL toluene at RT and

a water bath is then placed under the flask to cool down the reaction mixture at RT. The purification of CdSe QDs is performed by adding Ethanol (EtOH) in a 3:1 volume ratio relative to the reaction mixture. The resulting turbid solution is centrifuged three times at 13.5k rpm, 20 °C, for 15 minutes and the QDs pellet is finally redispersed in octane for further use and characterization. The size of as-synthesized CdSe QDs is optically determined as described by Jasieniak et al.[2]

c-ALD of CdSe@AlOx

Amorphous AlOx is grown around CdSe QDs by a modified procedure previously reported in our group.[3] Firstly, using a 10 mL vial with a septum cap, a transaminated aluminum precursor is prepared by adding a 1:1 molar ratio of tris(dimethylamido)aluminum(III) (TDMA-Al) and didodecylamine (dda) in toluene, so that a concentration of 0.05M could be achieved. The mixture is left stirring at 100 °C for 1 hour in a nitrogen-filled glovebox. A thin needle is placed in the septum cap to allow byproducts to be removed. After 1 hour, a transamidated aluminum precursor referred to as dda-TDMA-Al is obtained. It must be noted that the three native dimethylamine ligands are partially or completely replaced by dda ligands. We assume that a distribution of these products is obtained. To obtain CdSe@AlOx samples, a 4 mL vial is charged with a dilute colloidal dispersion of CdSe QDs and a small magnetic stirrer is placed inside. One complete c-ALD cycle consists of three steps: (1) dda-TDMA-Al is added in a 1:1 molar ratio with native oleate ligands on the surface of QDs and the solution is left stirring at RT for 15 minutes. (2) The temperature is raised to 70 °C and the solution is left stirring for 15 additional minutes to achieve a quantitative reaction of metal precursor with oleates. (3) OLAC is added in a 1:1 molar ratio with dda-TDMA-Al added in the first step, to get a final 1:1:1 oleates:dda-TDMA-Al:OLAC molar ratio. The solution is left stirring for 15 minutes in such a way that a complete layer of AlOx is grown around QDs surface. To obtain the photoactive CdSe@AlOx/9ACA nanocomposite, the same procedure is followed, but step (3) in the last cycle is modified: instead of adding OLAC only, a 1:1 mixture of 9ACA and OLAC in toluene is added in a 1:1 molar ratio with dda-TDMA-Al. If c-ALD is successfully performed, no precipitate should be observed during the process, indicating that the colloidal stability is retained and that no nucleation of the oxide occurred. All the samples are purified by adding EtOH in a 3:1 volume ratio. The resulting turbid solution is centrifuged at 7k rpm, 20 °C, for 10 minutes to remove any byproduct and excess of ligands. The pellets are then redispersed in octane for further characterization.

Preparation of CdSe QDs functionalized with 9ACA

To prepare CdSe QDs with 9ACA ligands bound to the surface, a simple ligand exchange is performed. Starting from the as-synthesized oleate-capped CdSe QDs a 0.03 M solution of 9ACA in toluene is prepared and a certain volume is added to CdSe QDs so that the desired ratio with native oleate ligands is obtained. To work in similar conditions to c-ALD, the solution is left stirring at 70 degrees in a nitrogen-filled glovebox for 20 minutes. Afterward, the solution is purified by adding a 3:1 volume ratio of EtOH and the resulting turbid solution is centrifuged at 7k rpm, at 20 °C, for 15 minutes. The CdSe/9ACA pellet is then redispersed in octane for further characterization. Due to the very low solubility of 9ACA in octane, all the molecules left in the solution are believed to be bound to the surface of QDs.

Calculation of average number of 9ACA per QD from UV-Vis spectra.

The average number of bound 9ACA molecules is calculated from UV-VIS absorption spectra as follows. First, the molar extinction coefficient at 360 nm of 9ACA is calculated using a solution with a known concentration of 9ACA in octane. The absorption spectra of CdSe@AlOx and CdSe@AlOx/9ACA for the same number of cycles are acquired after carefully washing the samples and the concentration of QDs is then calculated. The spectra are then normalized at the E_{1S} first absorption peak and the absorptions at 360 nm are subtracted. Because of the low solubility of 9ACA in octane, the absorption difference can be attributed to bound molecules. Knowing the molar extinction coefficient at 360 nm, the concentration of 9ACA is calculated employing the Lambert-Beer law. Finally, the average number of bound 9ACA per QD is found by dividing the concentration of 9ACA by that of QDs.

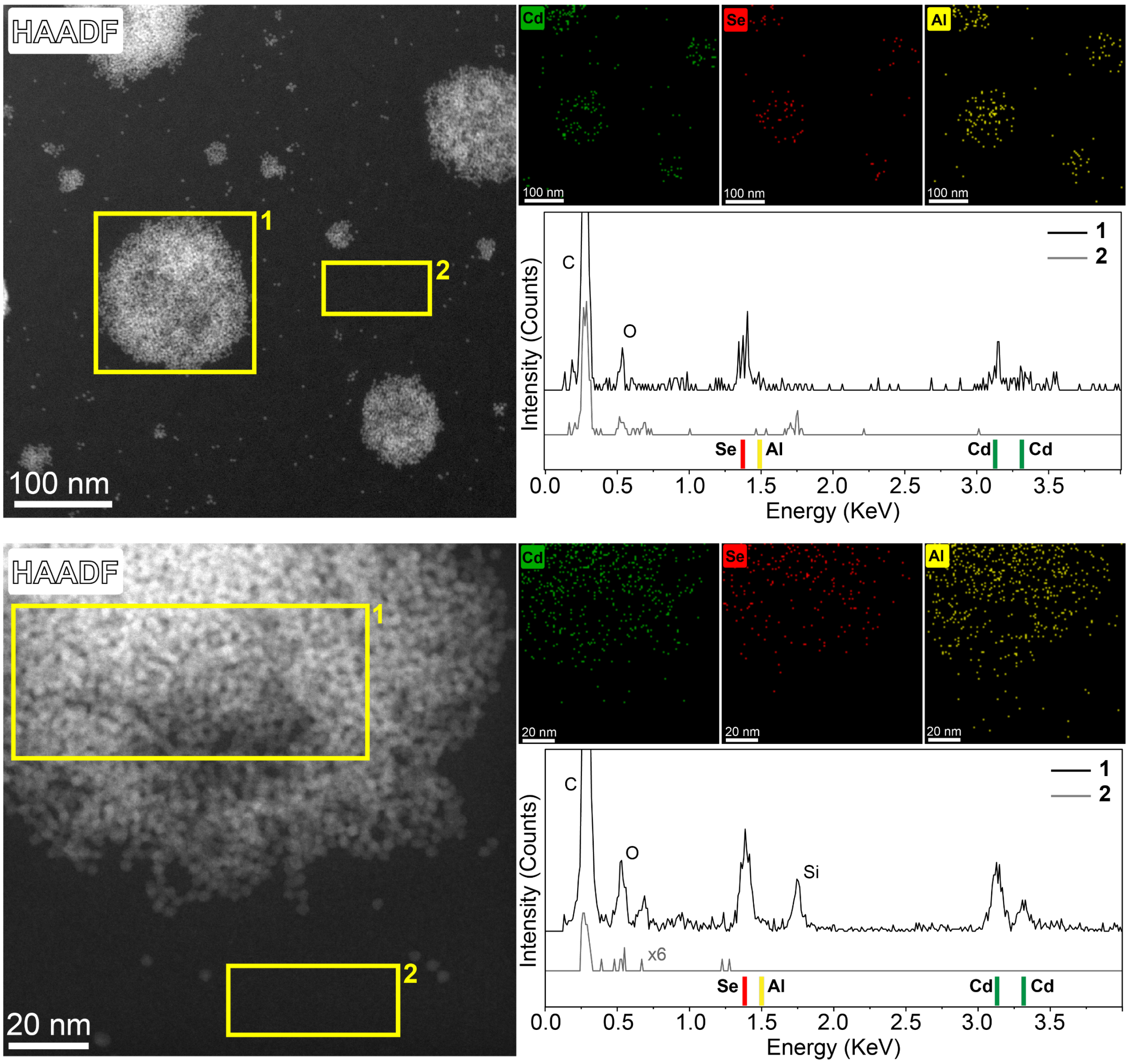

Figure S1: HAADF-STEM-EDXS of CdSe@AlOx n=10. Each image is supported by the EDX colored maps of Cd, Se and Al, along with the EDX spectra of the areas with and without CdSe@AlOx indicated by the yellow squares (1) and (2).

To have additional confirmation that the oxide shell solely grows around QDs, we acquired HAADF-STEM images and corresponding EDXS maps for a representative CdSe@AlOx sample where 10 c-ALD cycles were performed, and they are reported in Figure S1. HAADF-STEM images show two different portions of the grid. The corresponding EDX colored maps show a spatial correlation between Cd, Se and Al, suggesting that the oxide solely grows around the QDs with no evidence of homo-nucleation occurring during the process. The EDX spectra acquired in areas with and without CdSe@AlOx further confirm the presence of Al solely around QDs.

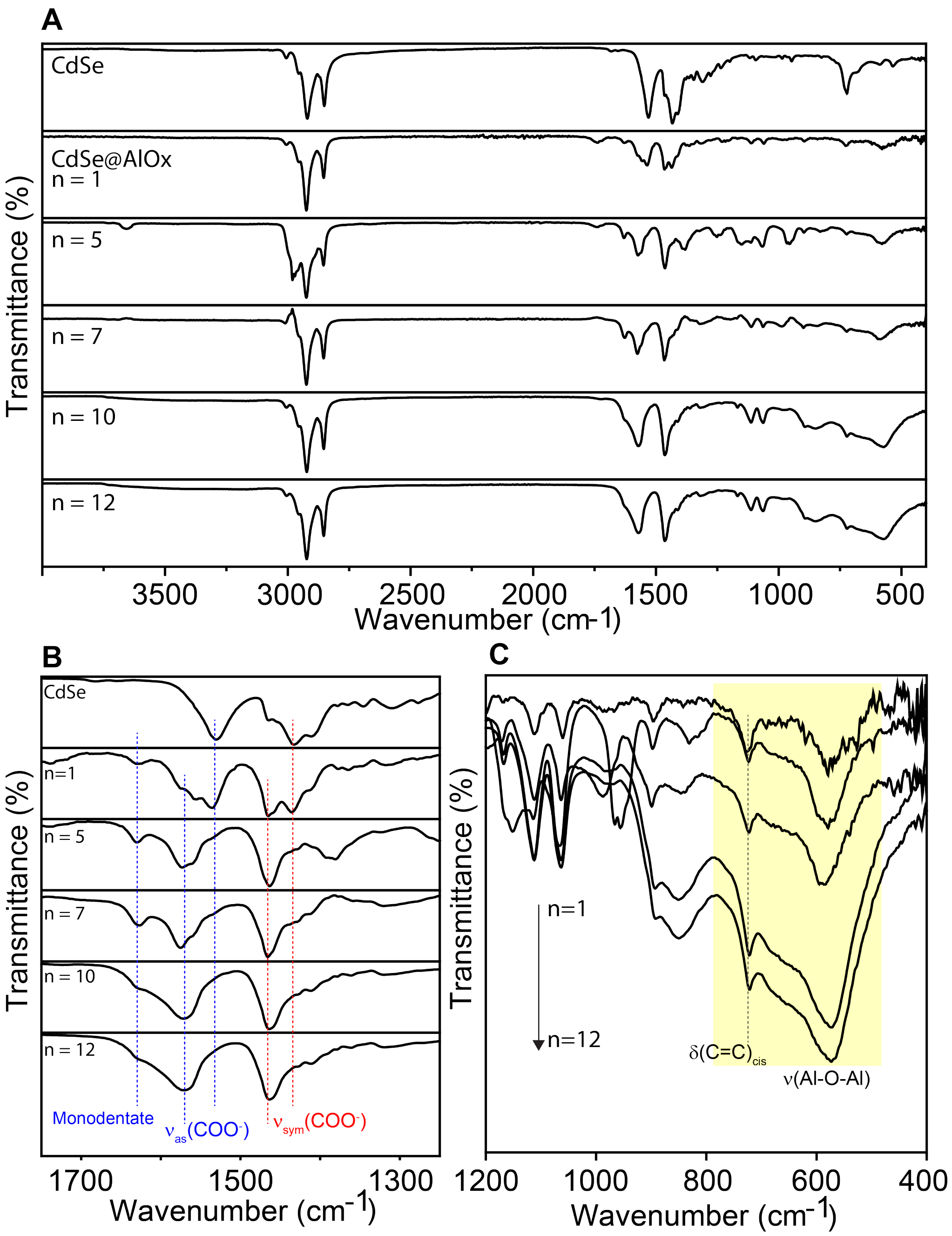

Figure S2: FTIR characterization of CdSe and CdSe@AlOx. (A) Full FTIR spectra of CdSe and CdSe@AlOx from 1 to 12 cycles. **(B)** Zoom in the 1300-1700 cm^{-1} region showing the COO^- symmetric and asymmetric stretching of bound oleates. **(C)** Zoom in the 400-1200 cm^{-1} region showing the Al-O-Al stretching.

Figure S2 shows the FTIR characterization of CdSe QDs and CdSe@AlOx from 1 to 12 cycles. The COO^- stretching of oleates between 1300 and 1700 cm-1 is reported in Figure S2-B. For

bare CdSe QDs, the difference between COO^- asymmetric and symmetric stretching is about 100 cm^{-1}, indicating a chelating configuration of oleates.[4,5] The same difference is found for all the CdSe@AlOx samples, suggesting that oleates are still mainly bound in a chelating geometry.However, both stretching modes are blue-shifted and a peak at around 1620 cm^{-1} also appears in the CdSe@AlOx samples. The shift at higher wavenumbers of the COO^- stretching is in line with a stronger coordination of carboxylic acids on the AlOx surface. Based on the Hard-Soft Acid-Base (HSAB) theory, the stronger Lewis acidity (i.e., higher charge density) of Al^{3+} compared to Cd^{2+} supports this hypothesis, suggesting that oleates may exhibit a stronger binding to the oxide shell than to the CdSe surface, thus showing a stretching at higher wavenumbers.[6] Indeed, stronger bonds require greater energies to vibrate; hence, we expect the vibration to appear at higher wavenumbers, i.e., higher frequencies. The conclusion of a stronger binding is also in agreement with reports on carboxylates on the surface of metal-oxide nanoparticles.[6,7] The shoulder centered at around 1620 cm^{-1} can be assigned to a portion of oleates in a monodentate configuration.[4,6,8,9] As n increases, this shoulder becomes broader, suggesting an increasing number of metal centers in a monodentate coordination with oleates. These observations suggest that oleate ligands on the c-ALD-grown metal oxide can exhibit different binding geometries, most likely chelating and monodentate. The 400-1200 cm^{-1} in Figure S2-C shows the Al-O-Al stretching centered at around 590 cm^{-1}. The appearance of the Al-O-Al stretching confirms the presence of alumina in all samples. The broadening and increase in intensity of the stretching qualitatively support the increase in Al content with the number of cycles, suggesting that the oxide is growing thicker.

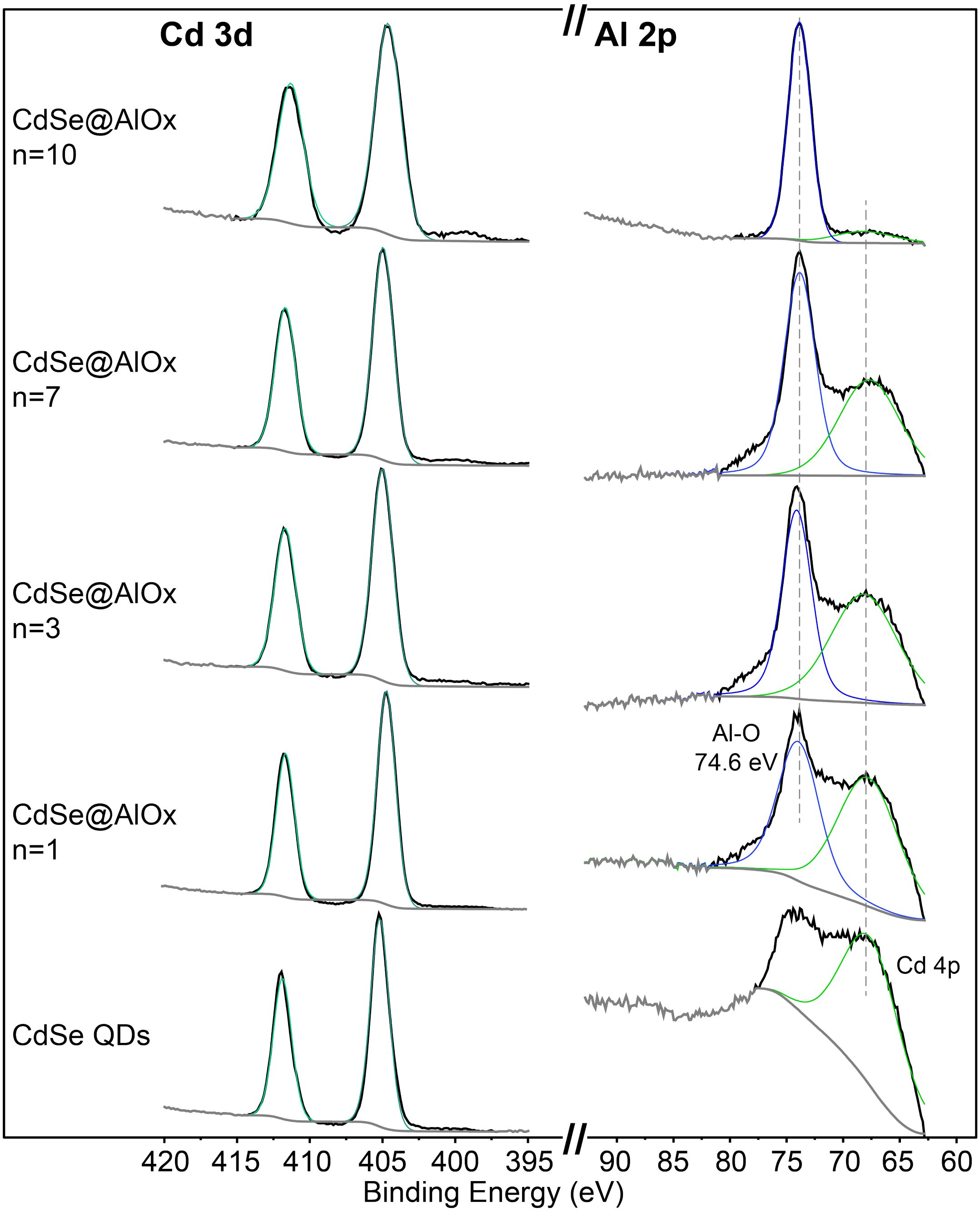

Figure S3: XPS characterization of CdSe and CdSe@AlOx. Cd 3d (left) and Al 2p (right) narrow-scan XPS spectra for CdSe QDs and CdSe@AlOx for n=1,3,7,10 cycles.

We performed XPS measurements to study the surface chemistry of CdSe QDs after the growth of AlOx of different thicknesses, for n=1,3,7,10. The narrow-scan XPS spectra of the Cd 3d region and Al 2p region are shown in Figure S3. CdSe and CdSe@AlOx samples show two peaks in the Cd 3d region (left), corresponding to Cd $3d_{3/2}$ and Cd $3d_{5/2}$ and centered at 411.8 and 405.2 eV, respectively, fitted with one component.[10] We note that the Cd $3d_{5/2}$ shifts to 404.9 eV in the presence of AlOx and the FWHM of both peaks increases with the number of

cycles, which can be explained by the greater contribution of the Cd-AlOx interface. However, no discernible Cd-O peak can be observed, indicating that the CdSe surface does not get oxidized. The Al 2p region (right) is used to qualitatively show the increase in Al content with the number of cycles. No attempt to resolve the spin-orbit splitting was done. For bare CdSe, the Cd 4p contribution can be found at 68.7 eV. A shoulder of the Cd 4p is present, which can be tentatively assigned to the contribution from surface Cd atoms. For all the CdSe@AlOx samples, the Cd 4p contribution is still present, but an additional peak appears at around 74.6 eV, which is expected for Al-O species. The Al-O contribution and its ratio with Cd 4p peak increase as the number of cycles increases, suggesting an increased Al content in the sample.

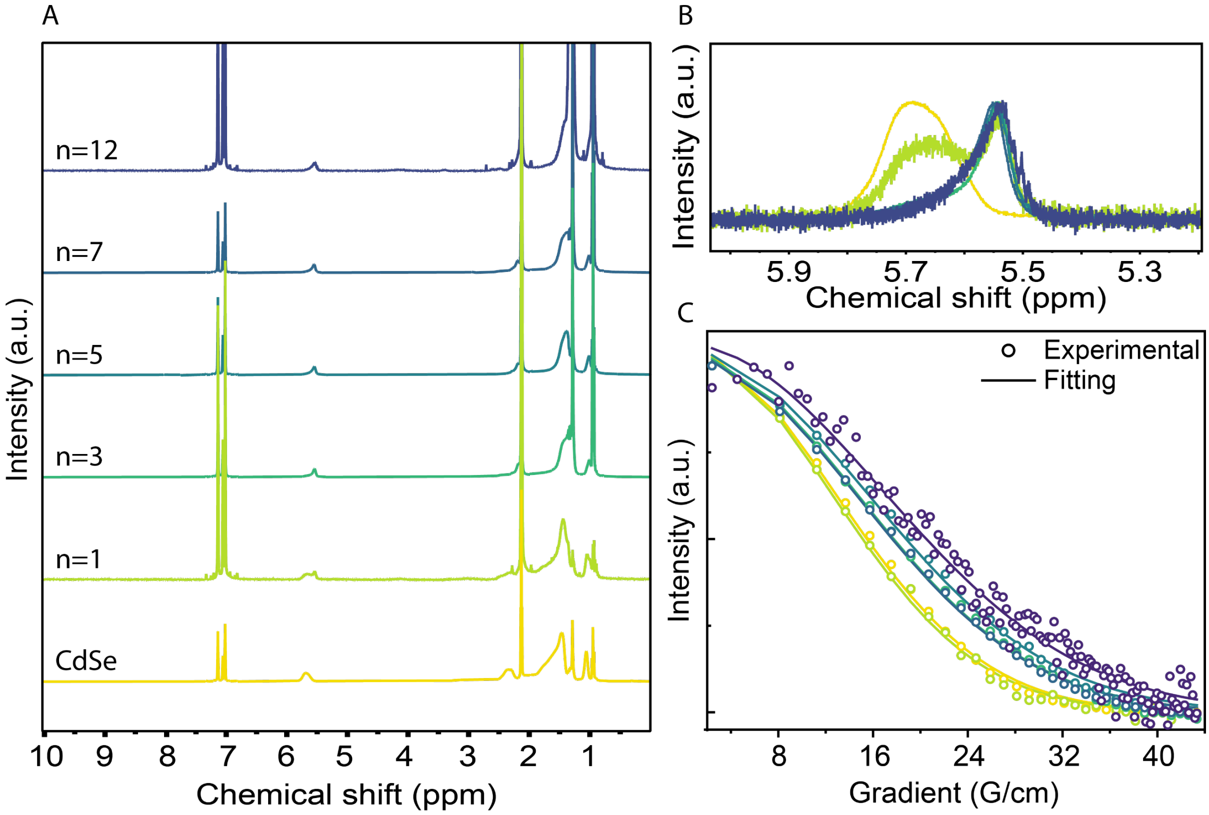

Figure S4: NMR characterization of CdSe and CdSe@AlOx QDs. (A) 1D-^{1}H NMR spectra of CdSe and CdSe@AlOx from 1 to 12 cycles. **(B)** Zoom in on the alkene resonance of bound oleates. **(C)** Attenuation curves and fitting of the intensity decay of the alkene resonance with eq. 3, as a function of gradient strength for each sample.

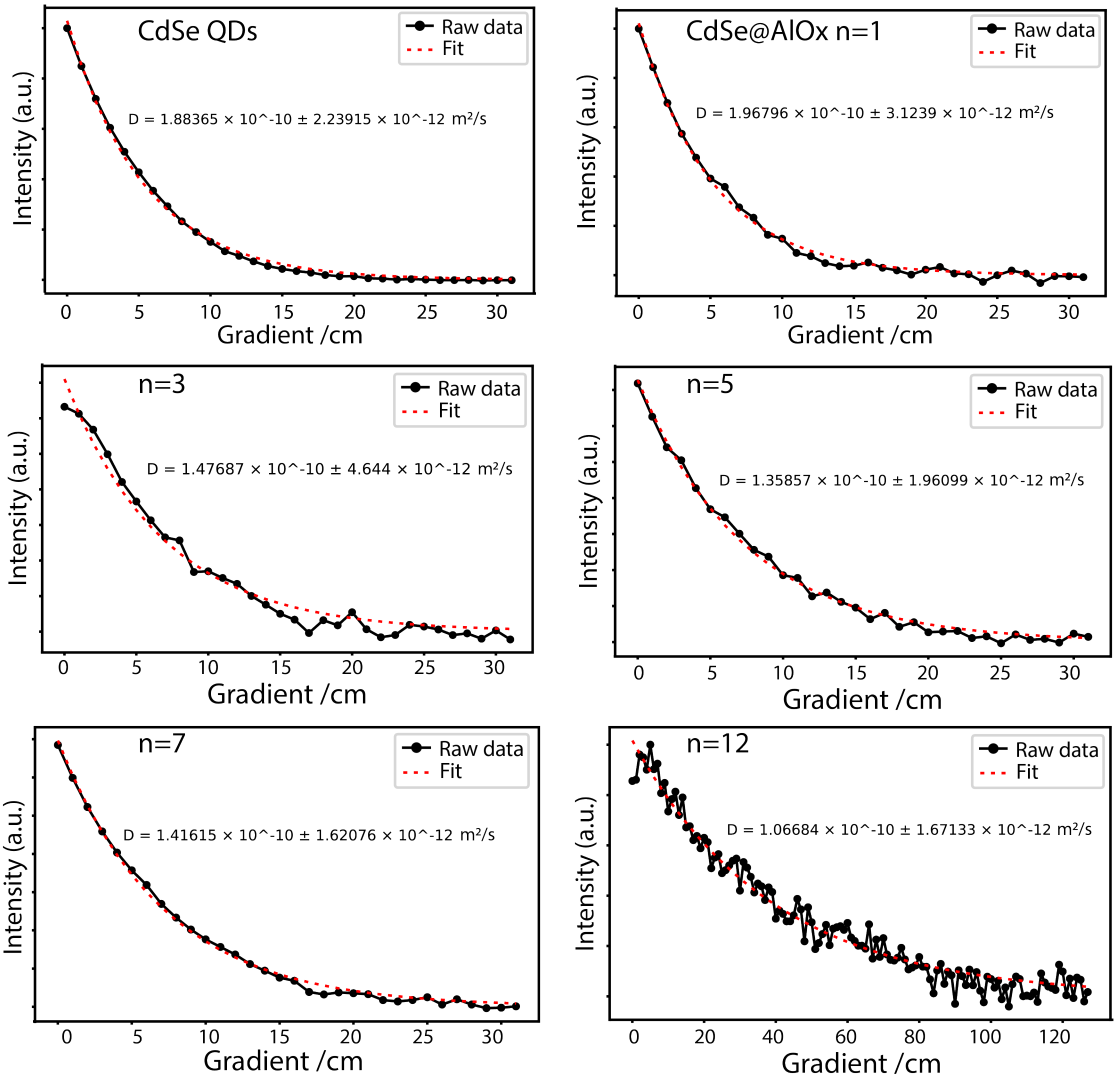

Figure S5: Results of the attenuation curve fitting for each sample investigated. We used 32 gradient steps for all the samples, except for n=12 where 128 gradient steps were used to obtain a better resolution. The diffusion coefficients with the respective standard deviation are provided.

The NMR characterization of CdSe QDs and CdSe@AlOx from 1 to 12 cycles is reported in Figure S4. The full spectra are reported in Figure S4-A. The alkene resonance of bound oleates (Figure S4-B) is used to track the evolution of the diffusion coefficients as the AlOx grows thicker. The alkene resonance of CdSe QDs (5.65 ppm) is upfield-shifted at around 5.55 ppm for all the CdSe@AlOx structures.[4–6] DOSY measurements confirm that both populations are bound to the QDs.[11,12] The 5.65 ppm resonance becomes less prominent when the AlOx grows around QDs. We attribute this observation to the different surface chemistry of metal oxide-coated CdSe and it is in line with previous reports on metal oxide nanocrystals.[6,8] In this case,

the n=1 sample displays both the 5.65 and 5.55 ppm resonances, indicating that some of the native oleates are not replaced by the metal precursor. DOSY measurements were performed to follow the trend of the diffusion coefficient as the AlOx grows thicker. The attenuation curves are shown in Figure S4-C and they were obtained by plotting the intensity of the 5.65 (for CdSe) and 5.55 ppm (for CdSe@AlOx) resonances as a function of the gradient strength, respectively. The decays are then fitted on TopSpin employing the Stejskal-Tanner equation (eq. 3)

$$I = I_0 \cdot \exp\left(-D(2\pi\gamma\delta)^2((\Delta - \frac{\delta}{3}) \cdot 10^4\right) \quad (3)$$

Where D is the diffusion coefficient, γ is the gyromagnetic ratio of ^{1}H (42.58 MHz/T), g is the gradient strength. The gradient strength is varied from 5 to 90%, while small delta (δ) and big delta (Δ) are set to 2.2 ms and 119.9 ms, respectively.

We obtained the diffusion coefficient at different n and we report the results of fittings in Figure S5, demonstrating that the up-field-shifted alkene resonance found for the CdSe@AlOx structures can be assigned to bound oleates on the oxide surface and not free oleic acid. We also tentatively fitted the decay with two components, but the fitting always yielded two similar diffusion coefficients, thus excluding the presence of free species with a larger diffusion coefficient. We also extracted the attenuation curves for CdSe@AlOx by fitting the intensity decay at 5.65 ppm. Again, the fitting resulted in diffusion coefficients comparable to the ones here reported, suggesting that they are anchored to the QDs surface.

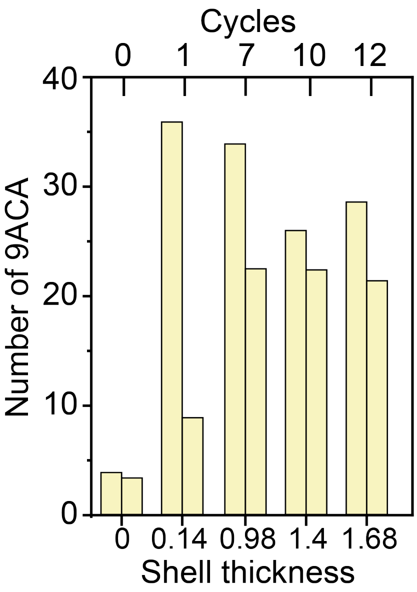

Figure S6: Average number of 9ACA molecules per QD at different shell thicknesses/cycles from UV-Vis spectra.

Two series of samples were prepared, indicated by two different columns, to check reproducibility. The shell thickness/cycle 0 corresponds to bare CdSe QDs where ligand exchange is performed with the same concentration of 9ACA used in the functionalization of the CdSe@AlOx samples. The data evidence that the ligand exchange is less efficient, which points at a stronger interaction of the 9ACA with the shell, either because of the stronger binding with the oxide or because of a covalent bonding forming in the Step II reported in Figure 1A.

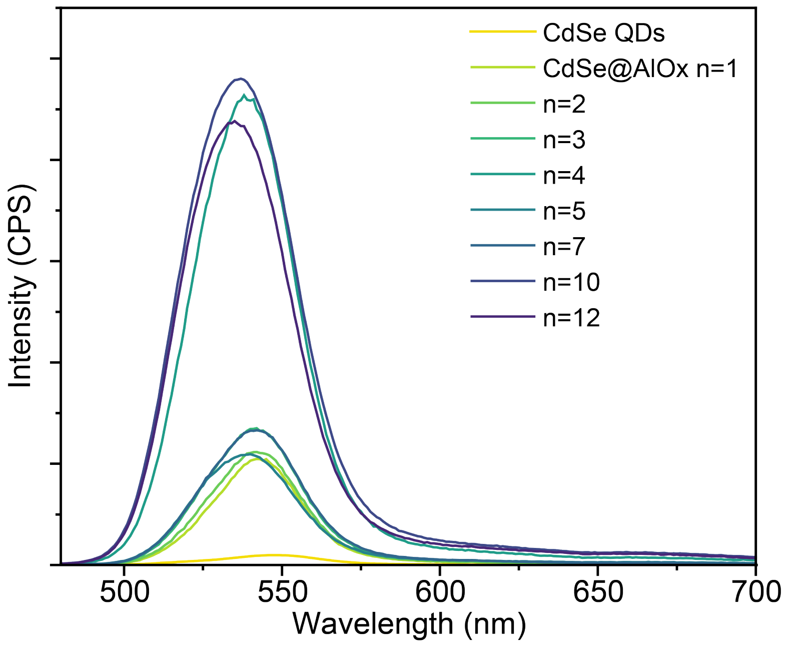

Figure S7: PL emission of CdSe QDs and CdSe@AlOx structures at different numbers of cycles normalized by the concentration of the corresponding sample.

Figure S7 shows the PL emission of CdSe and CdSe@AlOx at different numbers of cycles, normalized by the concentration of the corresponding sample. A higher PL band edge emission is observed for all CdSe@AlOx structures compared to as-synthesized CdSe QDs, suggesting that surface defect states are passivated by the growth of the oxide shell.

Interestingly, the most pronounced increase in the emission is observed for $n > 7$ which points towards an interesting optimal thickness of the shell to boost the emission.

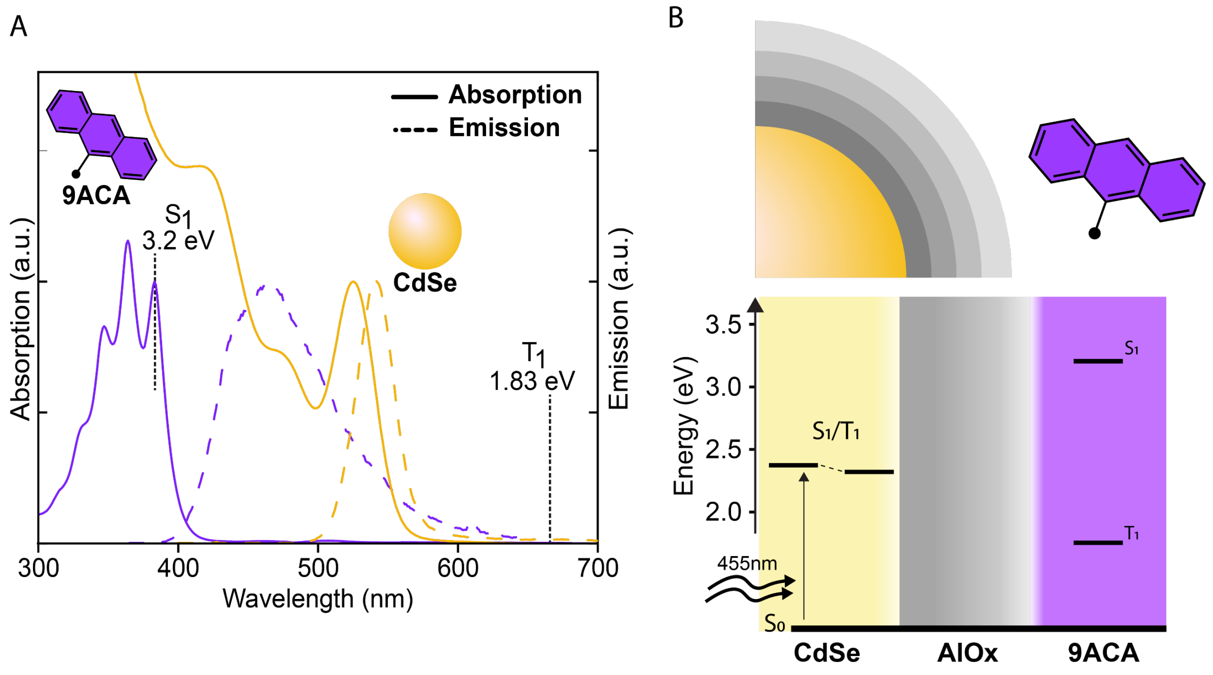

Figure S8: (A) Absorption and PL emission spectra of CdSe QDs and free 9ACA in octane. **(B)** Schematic representation of the CdSe@AlOx/9ACA donor-acceptor system (top) and corresponding energy diagram (bottom).

Figure S8 shows the absorption and emission spectra of CdSe QDs and free 9ACA, along with the energy alignment diagram. All spectra are acquired in octane after baseline correction. Figure S8-A shows the absorption and PL emission spectra of CdSe QDs (yellow) and 9ACA (violet) and the S_1 and T_1 excited states energy levels of 9ACA are provided. The spectra show no overlap between CdSe (donor) emission and 9ACA (acceptor) absorption. As the 9ACA S_1 energy can be estimated at around 3.2 eV, a Förster resonance energy transfer (FRET) can be ruled out.[14–16] Previous electrochemical studies also suggest that single-electron and hole transfers to anthracene ligands are thermodynamically unfavoured, hence, a net charged state (QD^+ - $9ACA^-$ or QD^- - $9ACA^+$) is not usually observed.[16–18] In similar systems, 9ACA sensitization typically occurs via a Dexter-like Triplet Energy Transfer (TEnT) that results in the population of the T_1 state (1.83 eV).[14,15,17,18] We, therefore, envision the CdSe exciton transfer to be only possible through a Dexter-like mechanism. A schematic of our donor-acceptor system and the corresponding energy diagram are provided in Figure S8-B. Upon excitation with 455 nm wavelength, CdSe is photoexcited. Owing to the ill-defined spin quantum number, the small S_1-T_1 splitting in CdSe allows for the population of the triplet-like exciton, which can then be transferred to the low-lying T_1 state of 9ACA via a Dexter-like mechanism. This transfer will result in the quenching of PL and PL decays of CdSe (Figure 2,

3 in the main text). When using CdSe@AlOx/9ACA, the amorphous AlOx will act as a dielectric spacer between the QD core and 9ACA, due to its wide band gap. The increase in the CdSe-9ACA separation with the shell thickness hints at a weaker electronic coupling and explains the observation of less effective quenching and smaller rates and efficiencies of TEnT.[19,20] Nonetheless, this hypothesis does not explain the observation of the distance-independent TEnT region beyond 1 nm. The unexpected distance-independent TEnT can most likely be explained by the presence of intrinsic defects within the oxide that act as intermediate states for the TEnT, as discussed in the main text.

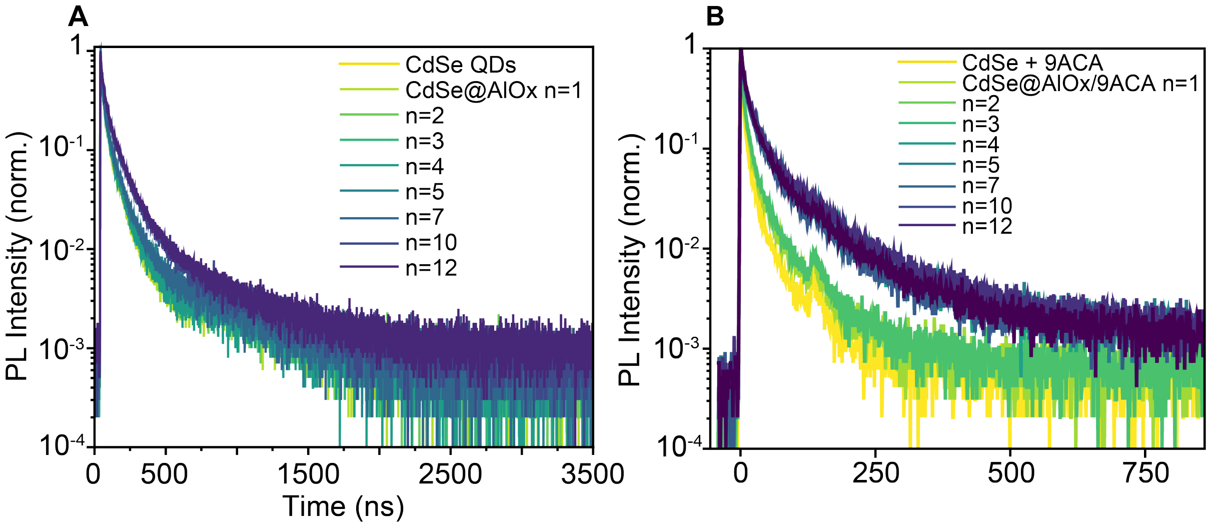

Figure S9: TRPL decays of CdSe and CdSe@AlOx **(A)** without 9ACA **(B)** and with 9ACA on a timescale of 3200 ns.

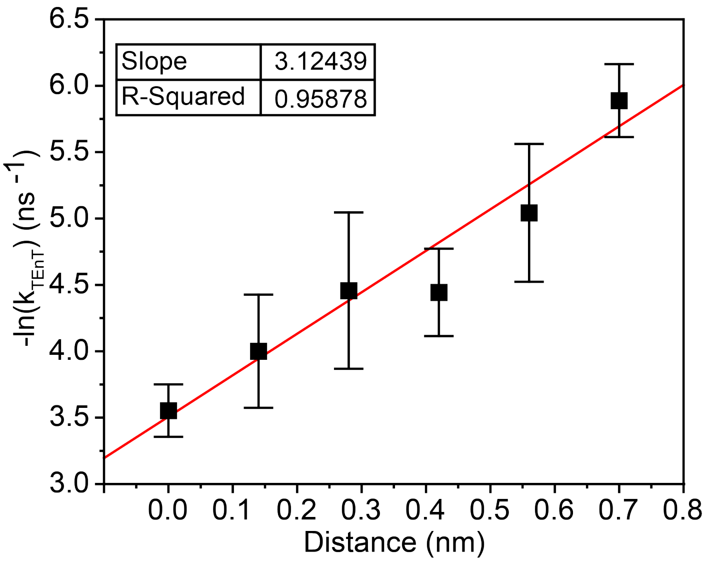

Figure S10: Fitting of the logarithmic rates of TEnT as a function of distance. The slope of the linear regression corresponds to the experimental damping coefficient β.

Figure S9 shows the TRPL decays of CdSe and CdSe@AlOx on a timescale of 3.2 µs without 9ACA (Figure S9-A) and with 9ACA (Figure S9-B). PL decays of CdSe@AlOx are

systematically longer compared to bare CdSe QDs as the shell grows thicker, suggesting the passivation of surface defect states and a longer-lived band-edge exciton. PL decays in the presence of 9ACA are systematically quenched compared to the corresponding sample without acceptor. The quenching is more evident within the first 800 ns, where most of the TEnT occurs. For this reason, the average lifetimes are calculated on a timescale of 800 ns following eq. 4.

$$\tau_{avg} = \frac{\sum_{i=1}^{n}(I_i \cdot t_i)}{\sum_{i=1}^{n}(I_i)} \tag{4}$$

No attempt was made to assign single lifetime components to specific decay pathways by an empirical multiexponential fitting, owing to the complexity of the system. The PL decays and PL decay quenching in the presence of 9ACA are modeled as TEnT, as we can exclude other exciton transfer pathways. To determine the rates of TEnT, K_{TEnT}, we employed eq. 5[21]

$$K_{TEnT} = \frac{1}{\langle\tau_{CdSe_n+9ACA}\rangle} - \frac{1}{\langle\tau_{CdSe_n}\rangle} \tag{5}$$

where $\langle\tau_{CdSe_n+9ACA}\rangle$ is the average lifetime of samples with bound 9ACA ligands and $\langle\tau_{CdSe_n}\rangle$ the average lifetime of samples without acceptors. In the absence of other decay pathways, the difference can be interpreted as the rate of TEnT. To correctly interpret the dynamics of TEnT, each CdSe@AlOx/9ACA sample is compared to the corresponding CdSe@AlOx where the same number of c-ALD cycles is performed. The efficiencies, Φ_{TEnT}, are then calculated using eq. 6.

$$\Phi_{TEnT}(\%) = \frac{K_{TEnT}}{1/\langle\tau_{CdSe_n+9ACA}\rangle} \cdot 100 \tag{6}$$

For a Dexter-like energy transfer, K_{TEnT} shows an exponential dependence from the donor-acceptor distance following eq. 7[19]

$$K_{TEnT} = K_0 \cdot e^{-\beta d} \tag{7}$$

Where K_0 is the pre-exponential factor and β is the damping coefficient, a measure of the electronic coupling exponential decay between CdSe QDs and 9ACA.[19,20] By fitting the K_{TEnT} for $n \leq 5$ with eq. 7, we experimentally found a damping coefficient β of 3.1 ± 0.3 nm^{-1}. The logarithmic rates of TEnT as a function of distance are plotted in Figure S10 and the results of fitting are provided. Recent advancements indicate that QDs may overcome the necessity for a strong electronic coupling required in single-step tunneling or super-exchange mechanisms of TEnT by favoring charge-transfer-mediated mechanisms, less demanding in terms of electronic

coupling.[18,19,22–25] For a classic tunneling mechanism, the β value is expected to be as high as the sum of that for two-electron transfer events.[20,26] In studies investigating TEnT from core@shell QDs, smaller coefficients are typically observed.[18,24] Particularly, Lai et al. reported a damping coefficient β of 6.2 nm^{-1} for CdSe@ZnS functionalized with 9ACA molecules, which is in agreement with an endothermic hole-transfer-mediated TEnT.[18] Similar values are reported when molecular conjugated bridges are used to separate the core from the acceptor ligand.[26,27]

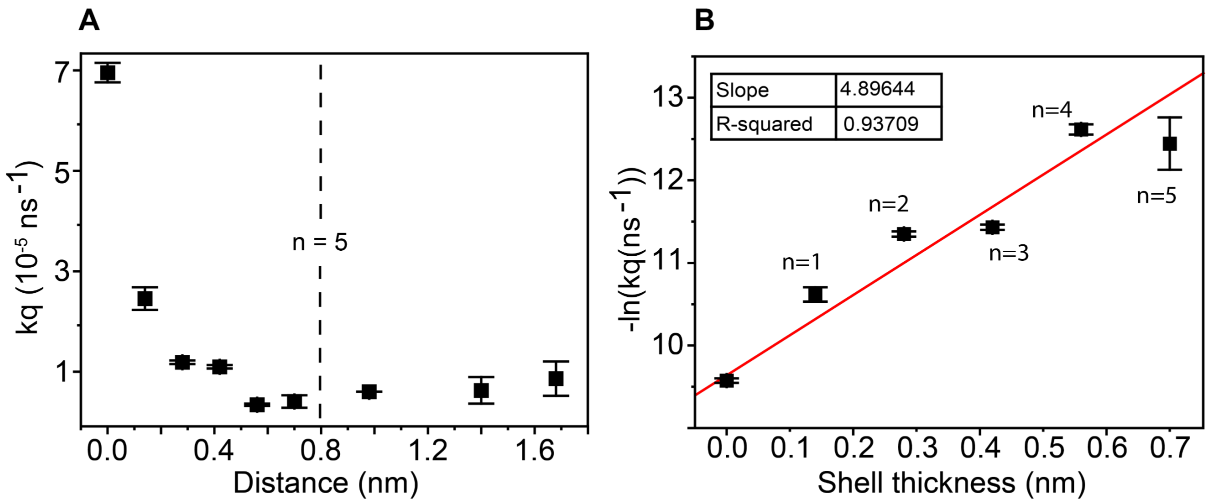

Figure S11: Results of the PL decay fitting using the Poisson-distributed population model. (A) Rate constants for TEnT (k_q) as a function of shell thickness (i.e., distance). **(B)** Natural logarithm of k_q as a function of shell thickness for $n \leq 5$ fitted with a line. The slope corresponds to the experimental damping coefficient β.

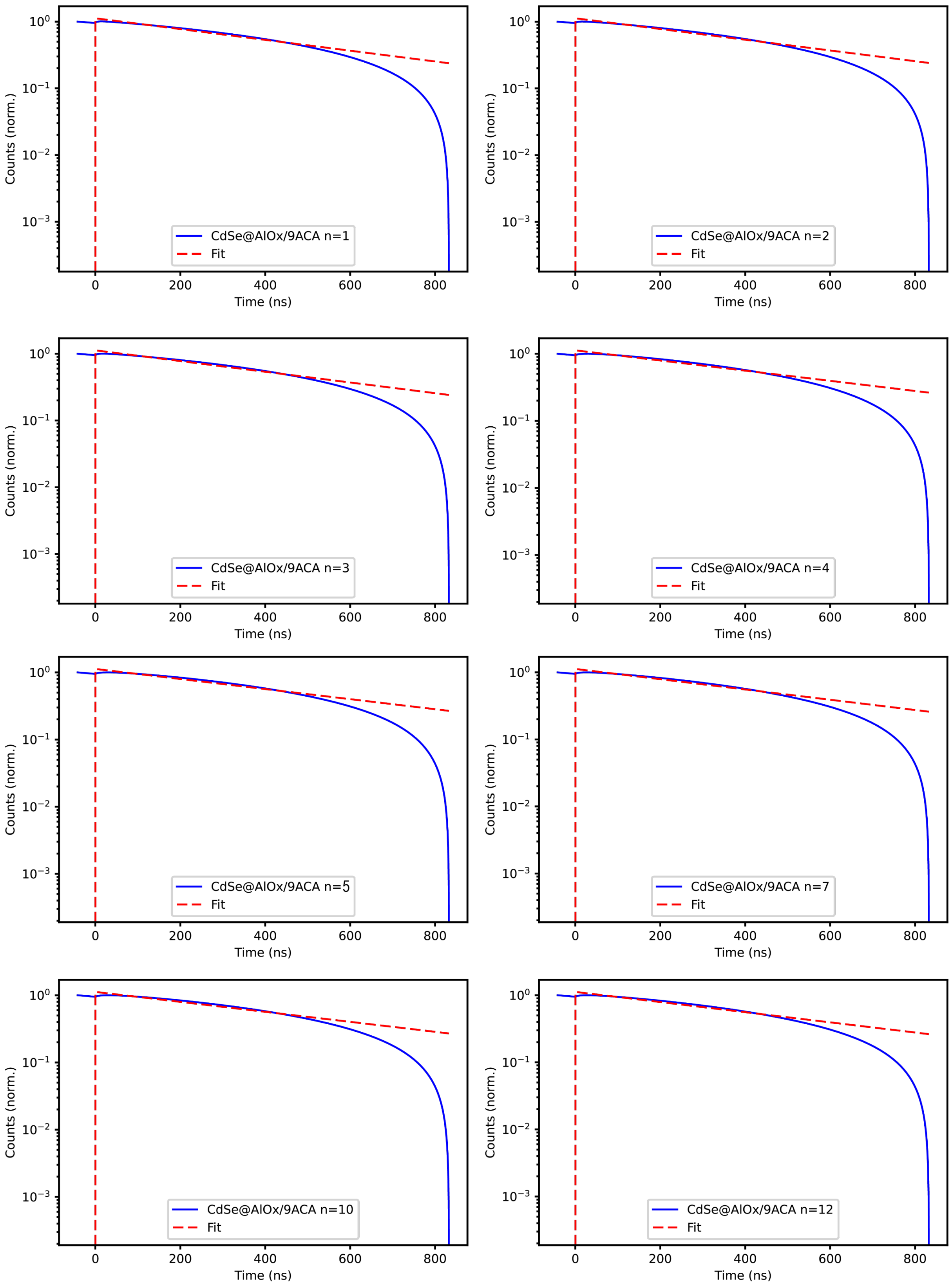

Figure S12: Fitting of integrative PL decays of CdSe@AlOx/9ACA with the Poisson-distributed population model for all the investigated shell thicknesses.

To extract the values of the rate constant of TEnT (k_q), we used a Poisson-distributed population model to analyze the TRPL data. We first integrated the PL decays to obtain population decays over time and then fit the integrated PL decays with eq. 8[14,27]

$$S(t) = S_0(t) \cdot \exp\left(-\mathrm{m}\left(1 - \exp\left(-\mathrm{K}_q \mathrm{t}\right)\right)\right) \tag{8}$$

Briefly, this model assumes there is a Poisson distribution of ACA acceptors on the surface of the QDs and assumes that the rate of triplet energy transfer for a single QD linearly scales with the number of bound acceptors. This model has been previously used for a variety of charge and energy transfer process involving semiconductor nanocrystals. In eq. 8, $S_0(t)$ and S(t) are the survival probabilities of the QD ensemble without and with 9ACA acceptors for the same shell thickness, respectively, m is the average number of bound 9ACA ,and k_q is the rate constant for TEnT which is defined as the rate of TEnT if each QD had exactly one bound ACA. The values of m were set from UV-vis absorption (Figure S6), making $S_0(t)$ and k_q the only adjustable parameters. To convert the observed PL decay traces to survival probabilities, we first cumulatively integrate the PL decays, which is a measure of the total number of decayed QDs after excitation given by $F(t) = \int_0^t f(t)dt$, where *f(t)* is the measured PL decay.[28] To convert the total number of decayed QDs to fraction of decayed QDs we normalize F(t) to obtain $F_{norm}(t)$. Lastly, we subtract the normalized integral from 1 to obtain the survival probability $S(t) = 1 - F_{norm}(t)$, i.e., the fraction of excited QDs at any given time. $S_0(t)$ is then fitted with a 3-exponential function: $S_0(t) = A_1 e^{-k_1 t} + A_2 e^{-k_2 t} + A_3 e^{-k_3 t}$. The amplitude and rate constants determined from the 3-exponential fit for each shell thickness are then held constant to fit S(t) to eq. 8 to determine k_q. The results are summarized in Figure S11. The Poisson model yields a similar trend to the model used in the main text (Figure 3), with rates of TEnT decreasing up to $n = 5$ and then remaining nearly constant for $n > 5$ (Figure S11-A). By employing eq. 4, we can also estimate the damping coefficient β for $n \leq 5$. The damping coefficient here found is 4.9 ± 0.6 nm^{-1} (Figure S11-B), which is slightly bigger than the value found with the main model (Figure S10), but still smaller than the expected values for a concerted two-electron tunneling mechanism and in agreement with a sequential hole-transfer-mediated mechanism.

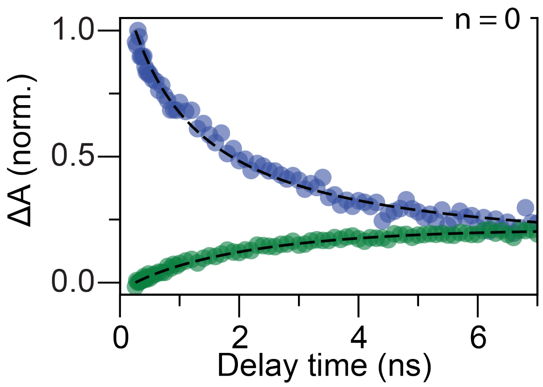

Figure S13: Ground state recovery at 515 nm (blue) and triplet formation at 430 nm (green) kinetics for CdSe QDs decorated with 9ACA ligands (n = 0).

The decay traces at 515 nm (blue) and 430 nm (green) characteristic of the QDs ground state bleach population (n_1) and ACA triplet population (n_2), respectively, are plotted as a function of delay time. At t = 0, n_1 is normalized to 1 and n_2 to 0. The evolution dynamics of both populations over time are fit (dashed lines) using the coupled differential equations:

$$\frac{dn_1}{dt} = -k_1 * n_1^2 - k_2 * n_1$$

$$\frac{dn_2}{dt} = -k_2 * n_1 - k_3 * n_2$$

With rate constants k_1, the ground-state recombination, k_2, the triplet formation and k_3, the triplet recombination. The extracted time constants are $1/k_1$ = 1.25 ns, $1/k_2$ = 8.5 ns, and $1/k_3$ = 8.2 ns.

References

(1) Flamee, S.; Cirillo, M.; Abe, S.; De Nolf, K.; Gomes, R.; Aubert, T.; Hens, Z. Fast, High Yield, and High Solid Loading Synthesis of Metal Selenide Nanocrystals. *Chem. Mater.* **2013**, *25* (12), 2476–2483. https://doi.org/10.1021/cm400799e.

(2) Jasieniak, J.; Smith, L.; Van Embden, J.; Mulvaney, P.; Califano, M. Re-Examination of the Size-Dependent Absorption Properties of CdSe Quantum Dots. *J. Phys. Chem. C* **2009**, *113* (45), 19468–19474. https://doi.org/10.1021/jp906827m.

(3) Green, P. B.; Segura Lecina, O.; Albertini, P. P.; Newton, M. A.; Kumar, K.; Boulanger, C.; Leemans, J.; Thompson, P. B. J.; Loiudice, A.; Buonsanti, R. Colloidal Atomic Layer Deposition on Nanocrystals Using Ligand-Modified Precursors. *J. Am. Chem. Soc.* **2024**, *146* (15), 10708–10715. https://doi.org/10.1021/jacs.4c00538.

(4) Kennehan, E. R.; Munson, K. T.; Doucette, G. S.; Marshall, A. R.; Beard, M. C.; Asbury, J. B. Dynamic Ligand Surface Chemistry of Excited PbS Quantum Dots. *J. Phys. Chem. Lett.* **2020**, *11* (6), 2291–2297. https://doi.org/10.1021/acs.jpclett.0c00539.

(5) Kennehan, E. R.; Munson, K. T.; Grieco, C.; Doucette, G. S.; Marshall, A. R.; Beard, M. C.; Asbury, J. B. Influence of Ligand Structure on Excited State Surface Chemistry of Lead Sulfide Quantum Dots. *J. Am. Chem. Soc.* **2021**, *143* (34), 13824–13834. https://doi.org/10.1021/jacs.1c06248.

(6) De Roo, J.; Justo, Y.; De Keukeleere, K.; Van den Broeck, F.; Martins, J. C.; Van Driessche, I.; Hens, Z. Carboxylic-Acid-Passivated Metal Oxide Nanocrystals: Ligand Exchange Characteristics of a New Binding Motif. *Angew Chem Int Ed* **2015**, *54* (22), 6488–6491. https://doi.org/10.1002/anie.201500965.

(7) Deblock, L.; Goossens, E.; Pokratath, R.; De Buysser, K.; De Roo, J. Mapping out the Aqueous Surface Chemistry of Metal Oxide Nanocrystals: Carboxylate, Phosphonate, and Catecholate Ligands. *JACS Au* **2022**, *2* (3), 711–722. https://doi.org/10.1021/jacsau.1c00565.

(8) De Roo, J.; Van Den Broeck, F.; De Keukeleere, K.; Martins, J. C.; Van Driessche, I.; Hens, Z. Unravelling the Surface Chemistry of Metal Oxide Nanocrystals, the Role of Acids and Bases. *J. Am. Chem. Soc.* **2014**, *136* (27), 9650–9657. https://doi.org/10.1021/ja5032979.

(9) Zhang, J.; Zhang, H.; Cao, W.; Pang, Z.; Li, J.; Shu, Y.; Zhu, C.; Kong, X.; Wang, L.; Peng, X. Identification of Facet-Dependent Coordination Structures of Carboxylate Ligands on CdSe Nanocrystals. *J. Am. Chem. Soc.* **2019**, *141* (39), 15675–15683. https://doi.org/10.1021/jacs.9b07836.

(10) Segura Lecina, O.; Hope, M. A.; Venkatesh, A.; Björgvinsdóttir, S.; Rossi, K.; Loiudice, A.; Emsley, L.; Buonsanti, R. Colloidal-ALD-Grown Hybrid Shells Nucleate via a Ligand–Precursor Complex. *J. Am. Chem. Soc.* **2022**, *144* (9), 3998–4008. https://doi.org/10.1021/jacs.1c12538.

(11) Hens, Z.; Martins, J. C. A Solution NMR Toolbox for Characterizing the Surface Chemistry of Colloidal Nanocrystals. *Chem. Mater.* **2013**, *25* (8), 1211–1221. https://doi.org/10.1021/cm303361s.

(12) Hens, Z. Ligands on Nanocrystal Surfaces, the ^{1} H Nuclear Magnetic Resonance Fingerprint. *Acc. Chem. Res.* **2023**, *56* (12), 1623–1633. https://doi.org/10.1021/acs.accounts.3c00170.

(13) De Roo, J.; Yazdani, N.; Drijvers, E.; Lauria, A.; Maes, J.; Owen, J. S.; Van Driessche, I.; Niederberger, M.; Wood, V.; Martins, J. C.; Infante, I.; Hens, Z. Probing Solvent–Ligand Interactions in Colloidal Nanocrystals by the NMR Line Broadening. *Chem. Mater.* **2018**, *30* (15), 5485–5492. https://doi.org/10.1021/acs.chemmater.8b02523.

(14) Piland, G. B.; Huang, Z.; Lee Tang, M.; Bardeen, C. J. Dynamics of Energy Transfer from CdSe Nanocrystals to Triplet States of Anthracene Ligand Molecules. *J. Phys. Chem. C* **2016**, *120* (11), 5883–5889. https://doi.org/10.1021/acs.jpcc.5b12021.
(15) Montalti, M.; Credi, A.; Prodi, L.; Gandolfi, M. T. Handbook of Photochemistry.
(16) Tannaci, J. F.; Noji, M.; McBee, J.; Tilley, T. D. 9,10-Dichlorooctafluoroanthracene as a Building Block for n-Type Organic Semiconductors. *J. Org. Chem.* **2007**, *72* (15), 5567–5573. https://doi.org/10.1021/jo070404a.
(17) Mongin, C.; Garakyaraghi, S.; Razgoniaeva, N.; Zamkov, M.; Castellano, F. N. Direct Observation of Triplet Energy Transfer from Semiconductor Nanocrystals. *Science* **2016**, *351* (6271), 369–372. https://doi.org/10.1126/science.aad6378.
(18) Lai, R.; Liu, Y.; Luo, X.; Chen, L.; Han, Y.; Lv, M.; Liang, G.; Chen, J.; Zhang, C.; Di, D.; Scholes, G. D.; Castellano, F. N.; Wu, K. Shallow Distance-Dependent Triplet Energy Migration Mediated by Endothermic Charge-Transfer. *Nat Commun* **2021**, *12* (1), 1532. https://doi.org/10.1038/s41467-021-21561-1.
(19) You, Z.; Hsu, C. Theory and Calculation for the Electronic Coupling in Excitation Energy Transfer. *Int. J. Quantum Chem.* **2014**, *114* (2), 102–115. https://doi.org/10.1002/qua.24528.
(20) You, Z.-Q.; Hsu, C.-P.; Fleming, G. R. Triplet-Triplet Energy-Transfer Coupling: Theory and Calculation. *The Journal of Chemical Physics* **2006**, *124* (4), 044506. https://doi.org/10.1063/1.2155433.
(21) Jin, T.; Lian, T. Trap State Mediated Triplet Energy Transfer from CdSe Quantum Dots to Molecular Acceptors. *The Journal of Chemical Physics* **2020**, *153* (7), 074703. https://doi.org/10.1063/5.0022061.
(22) Luo, X.; Liang, G.; Han, Y.; Li, Y.; Ding, T.; He, S.; Liu, X.; Wu, K. Triplet Energy Transfer from Perovskite Nanocrystals Mediated by Electron Transfer. *J. Am. Chem. Soc.* **2020**, *142* (25), 11270–11278. https://doi.org/10.1021/jacs.0c04583.
(23) Hasham, M.; Narayanan, P.; Yarur Villanueva, F.; Green, P. B.; Imperiale, C. J.; Wilson, M. W. B. Sequential Carrier Transfer Can Accelerate Triplet Energy Transfer from Functionalized CdSe Nanocrystals. *J. Phys. Chem. Lett.* **2023**, *14* (7), 1899–1909. https://doi.org/10.1021/acs.jpclett.2c03443.
(24) Jia, Y.; Chen, J.; Wu, K.; Kaledin, A.; Musaev, D. G.; Xie, Z.; Lian, T. Enhancing Photo-Reduction Quantum Efficiency Using Quasi-Type II Core/Shell Quantum Dots. *Chem. Sci.* **2016**, *7* (7), 4125–4133. https://doi.org/10.1039/C6SC00192K.
(25) Vura-Weis, J.; Abdelwahed, S. H.; Shukla, R.; Rathore, R.; Ratner, M. A.; Wasielewski, M. R. Crossover from Single-Step Tunneling to Multistep Hopping for Molecular Triplet Energy Transfer. *Science* **2010**, *328* (5985), 1547–1550. https://doi.org/10.1126/science.1189354.
(26) Huang, Z.; Xu, Z.; Huang, T.; Gray, V.; Moth-Poulsen, K.; Lian, T.; Tang, M. L. Evolution from Tunneling to Hopping Mediated Triplet Energy Transfer from Quantum Dots to Molecules. *J. Am. Chem. Soc.* **2020**, *142* (41), 17581–17588. https://doi.org/10.1021/jacs.0c07727.
(27) Li, X.; Huang, Z.; Zavala, R.; Tang, M. L. Distance-Dependent Triplet Energy Transfer between CdSe Nanocrystals and Surface Bound Anthracene. *J. Phys. Chem. Lett.* **2016**, *7* (11), 1955–1959. https://doi.org/10.1021/acs.jpclett.6b00761.
(28) Van Driel, A. F.; Nikolaev, I. S.; Vergeer, P.; Lodahl, P.; Vanmaekelbergh, D.; Vos, W. L. Statistical Analysis of Time-Resolved Emission from Ensembles of Semiconductor Quantum Dots: Interpretation of Exponential Decay Models. *Phys. Rev. B* **2007**, *75* (3), 035329. https://doi.org/10.1103/PhysRevB.75.035329.
